# Supplementary material for: ChemRAP uncovers specific mRNA translation regulation via RNA 5′ phospho-methylation
Source: EMBO Rep. 2024 Jan 23;25(3):1570–88. doi: 10.1038/s44319-024-00059-z (PMC10933402; doi:10.1038/s44319-024-00059-z)
Supplement: Supplementary file 1 — Appendix [file 44319_2024_59_MOESM1_ESM.pdf]

APPENDIX

Appendix Table S1 .... Page 2
Appendix Table S2 .... Page 3
Appendix Table S3 .... Page 4
Appendix Table S4 .... Page 5
Appendix Figure S1 .... Page 6
Appendix Figure S2 .... Page 7
Appendix Figure S3 .... Page 8
Appendix Figure S4 .... Page 9
Appendix Figure S5 .... Page 10
Appendix Figure S6 .... Page 12
Appendix Figure S7 .... Page 13
Appendix Figure S8 .... Page 14
Appendix Figure S9 .... Page 15
Appendix References .... Page 16

Appendix Table S1. mRNAs reads in iCLIP-seq.

Gene	Control α GFP	Control_ α EPRS	BCDIN3D-KO α GFP	BCDIN3D-KO α EPRS	Position of EPRS foot-print
MTMR1	1	119562	0	0	Coding exon
CANX	0	72757	0	0	Coding exon
JUP	0	68150	0	0	Coding exon
CEP164	0	55132	1	0	Coding exon
LRPPRC	0	45501	0	0	5'UTR / Coding exon
RMND5A	0	0	0	26503	3'UTR
PGK1	0.08	21746.67	0	0	5'UTR / Coding exon
MTIF2	0	0	0	18919.5	Coding exon
NRG4	0	0	0	14147	intron/AluJr
XRN1	0	9694	0	0	3'UTR
LSM12	0	8603.5	0	0	
DHFR	0	0	0.25	6981.56	Intron
JARID2	0	0	0	5575	Coding exon
DAG1	0	0	0	2841	3'UTR
FASN	0	0	0	1861	Coding exon
CRIP1	0	0	0	1455	5'UTR
RPL7A	0.25	0	0.12	500	3'UTR

Appendix Table S2. RNA-seq results in HeLa-S3-FlpIn- control and BCDIN3D-KO cells of the mRNAs with EPRS iCLIP sites. See Dataset EV3 for the full RNA-seq results.

Gene	baseMean	log2Fold-Change	p-value	p-adj
MTMR1	1751.33349	0.03064416	0.68684696	0.92178219
CANX	38228.0262	0.12070579	0.09624541	0.39960118
JUP	5554.49954	-0.3310084	1.28E-05	0.00049256
CEP164	1255.35835	0.31060454	0.00193129	0.02704963
LRPPRC	11983.7898	0.01757609	0.78956905	0.95736604
RMND5A	4423.29622	-0.1370109	0.05545555	0.28968221
PGK1	53729.9814	-0.0850023	0.61697046	0.8940534
MTIF2	3323.16288	-0.0299437	0.68176348	0.92060709
NRG4	633.716284	-0.2158256	0.05428802	0.28580746
XRN1	1759.23033	-0.0898103	0.33283306	0.72264125
LSM12	296.564927	0.13878374	0.37113679	0.75577725
MTRNR2L8	0.61040678	-1.4203329	0.67712804	NA
MTRNR2L12	0.98725097	2.0803548	0.41492304	NA
JARID2	944.445754	0.12334983	0.20708461	0.59042069
DAG1	8296.13841	0.02345874	0.69110551	0.92338903
FASN	24367.2266	0.19936486	0.22463226	0.61115227
CRIP1	5776.36664	-0.211977	0.00343379	0.0425433
RPL7A	62633.1877	-0.0050119	0.93156071	0.98776417

Appendix Table S3. List of antibodies.

Antibody	Provider	Catalog number	Lot number
Anti-GFP	Invitrogen	A11122	779558
Anti-BCDIN3D	Sigma	HPA039911	R36480
Anti-MARS (Western Blots)	Abcam	ab50793	GR63568-7
Anti-MARS (Immunofluorescence)	Sigma	SAB1409302-50UG	11140
Anti-EPRS	Abcam	ab31531	GR211593-1
Anti-EPRS (Immunoprecipitation)	Bethyl Laboratories	A303-959A	A303-959A-1
Anti-LRPPRC	Santa Cruz	sc-166178	K1918
Anti-RPS6	Cell Signaling	#2217	
Anti-PGK1	Santa Cruz	sc-130335	J1620
Anti-Ago2	Sigma	SAB4200085	# 2
Anti-αTubulin	Cell Signaling	#3873s	8
Anti-βTubulin	Cell Signaling	#2128	7
Anti-ATPIF1	Invitrogen	Ab21355	1662872
Anti-HSP60	Santa Cruz	sc-59567	

Appendix Table S4. List of primers and siRNAs.

Primers	Code	Sequence
ALAS1_ FWD	BX00060	CCTTGGCCTTAGCAGTTTTG
ALAS1_ REV	BX00061	CCAAGATGATGGAAGTTGGG
B2M_FWD	BX00062	AATGTCGGATGGATGAAACC
B2M_REV	BX00063	TCTCTCTTTCTGGCCTGGAG
LRPPRC_FWD#1	BX00936	CGCTGCGGGGACGTTCGAGCA
LRPPRC_REV#1	BX00937	CCTGGCTGGGCTCAGTAGTC
LRPPRC_FWD#2	BX00961	GCCCCGTTCGGAAATTTTC
LRPPRC_REV#2	BX00962	CGGAGGACTACTGAGCCCA
PGK1_FWD	BX00907	TTTCCAAAATGTCGCTTTCTAAC
PGK1_REV	BX00908	GACCCGCTTCCCTTTAACGTC
LRPPRC RNA#1	RNA-BX00061	5'P CUUCUGGCGGAGCGUGCUUCCCGCUGCGG GGACGUUCGAGCA
LRPPRC RNA#2	RNA-BX00062	5'P CUUCUGGCGGAGCGUGCUUCCCGCUGCGG GGACGUUCGAGCAAUGGCAGCCCUGCUGA GAUCC
siLRPPRC	RNA-BX-63	Sense: AAAUGGAUGUCUGUCUGAUAGUGAU[dT][dT] Antisense: [Phos]AUCACUAUCAGACAGACAUCCAUUU[dT][dT]
siNC	Dharmacon	ON-TARGETplus Non Targeting Pool (D-001810-10-05)
siBCDIN3D	Dharmacon	ON-TARGETplus (L-018768-02-0005)

Appendix Figures:

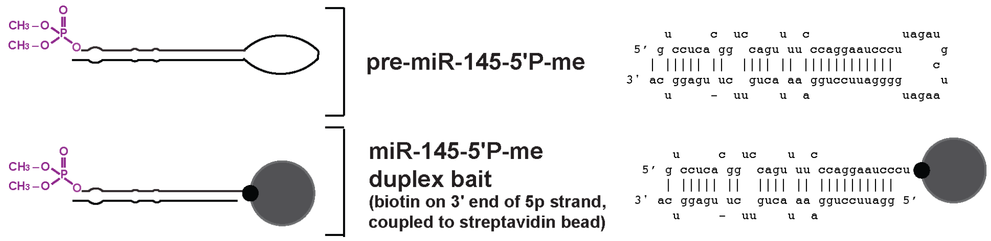

Appendix Fig S1. Schematic representation of pre-miR-145 and the miR-145 duplex used as bait in the ChemRAP experiments.

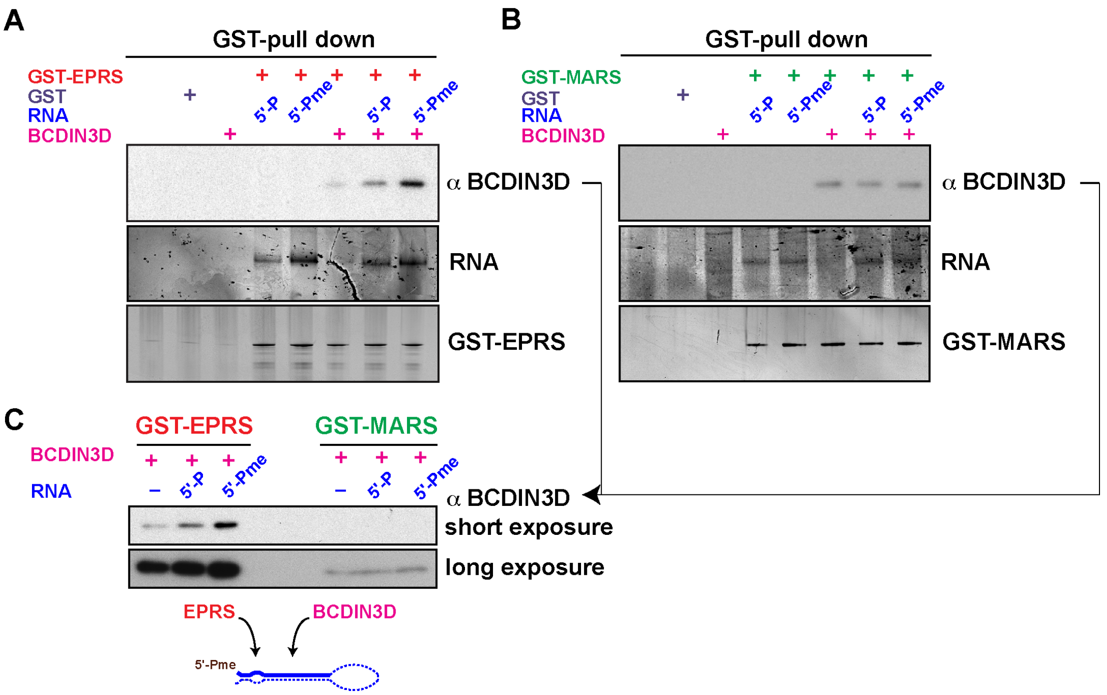

Appendix Fig S2. BCDIN3D and EPRS interact through a 5'Pme RNA *in vitro*.

(A) GST pull-down with GST-EPRS assessing binding of untagged recombinant BCDIN3D in the absence or presence of pre-miR-145 5'-P or 5'P-me.

(B) GST pull-down with GST-MARS assessing binding of BCDIN3D as in (**A**).

(C) Direct comparison of BCDIN3D binding to GST-EPRS and GST-MARS. The top panel is the same as in Fig 3B.

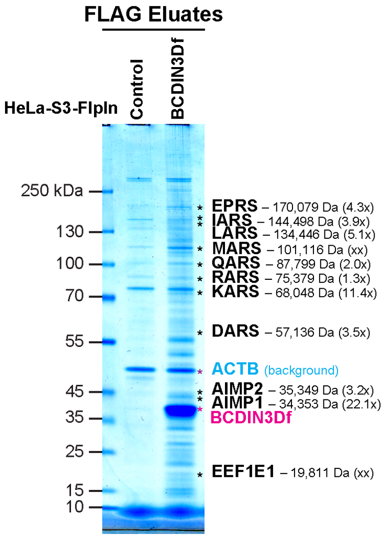

Appendix Fig S3. BCDIN3D interacts with MSC subunits in cells.

Image of a representative Bis-Tris 4-12% PAGE gel loaded with 10 µL of HeLa-S3-Flp-In-Control and -BCDIN3Df FLAG eluates and stained with Colloidal Coomassie. Black asterisks show the bands of the MSC subunits, their predicted molecular weight in Daltons, as well as the ratios of BCDIN3Df/Control of their iBAQ values normalized to ACTB background protein in the LC-MS/MS analysis of the shown samples (xx indicates that a ratio could not be determined because the iBAQ value obtained in the control samples was 0). Colored asterisks show the ACTB (top background band) and BCDIN3Df.

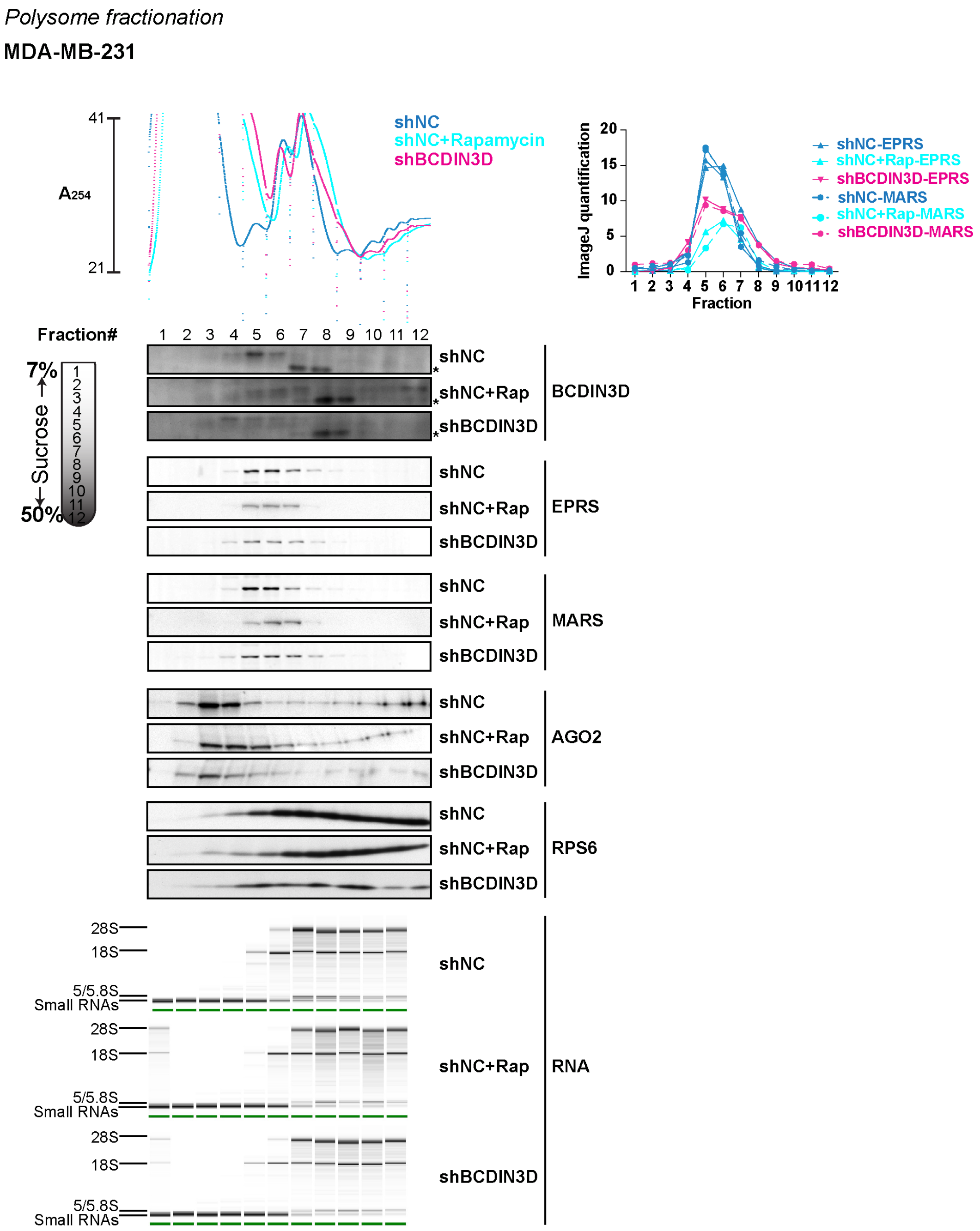

Appendix Fig S4. BCDIN3D depletion perturbs EPRS and MARS sedimentation in polysome fractionation in a similar manner to mTOR inhibition with Rapamycin.

Polysome lysates from the indicated MDA-MB-231 cells were fractionated on a 7-50% sucrose gradient and shown are from top to bottom: the real time recording of OD_{254}; western blots with the indicated antibodies of 20 µl of each fraction (quantification of EPRS and MARS western blots is shown on the top right graph); Bioanalyzer analysis of RNAs purified from each fraction. Note that the 40S peak in the shNC+Rap and shBCDIN3D samples is masked by the soluble fraction, as previously observed during polysome fractionation in human cells (Ceci *et al*, 2003). The asterisk indicates a non-specific band detected by the BCDIN3D antibody.

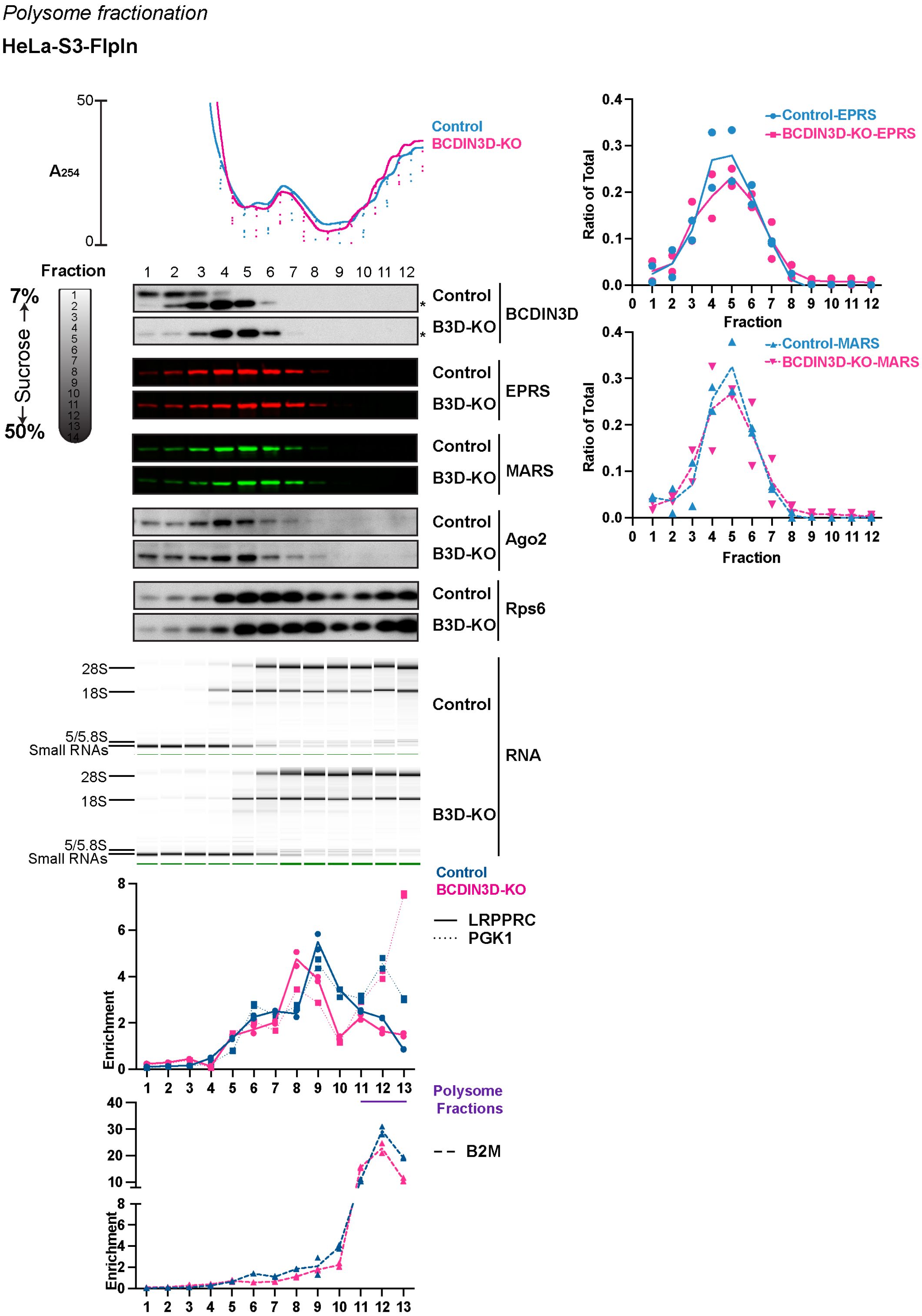

Appendix Fig S5 | BCDIN3D knock-out does not affect global translation and EPRS and MARS sedimentation in polysome fractionation in HeLa-S3-FlpIn cells.

Polysome lysates from HeLa-S3-FlpIn control and BCDIN3D-KO cells were fractionated on a 7-50% sucrose gradient and shown are from top to bottom:

The real time recording of OD_{254};

Western blots with the indicated antibodies of 20 μL of each fraction. Quantification of EPRS and MARS western blots is shown on the top right graphs, shown is mean from 2 independent biological repeats. The asterisk indicates a non-specific band detected by the BCDIN3D antibody.

Bioanalyzer analysis of RNAs purified from each fraction.

RTqPCR analysis of LRPPRC, PGK1, and B2M mRNA from each fraction of the same polysome fractionations (shown is mean from n=2 technical replicates). Normalization was done over the average Ct of each mRNA, which did not show significant differences in control and BCDIN3D-KO cells.

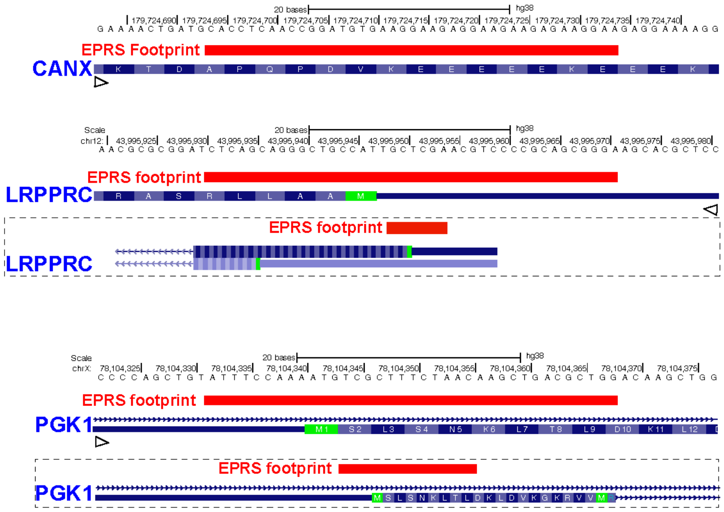

Appendix Fig S6. Examples of EPRS footprints in iCLIP-seq data.

iCLIP-seq EPRS footprints on the CANX, LRPPRC and PGK1 mRNAs shown on UCSC genome browser (hg38). For each example, shown are: the scale, the position on the chromosome, the DNA sequence of the Watson strand (note that the coding sequence of LRPPRC gene is on the Crick strand), the EPRS footprint, the representation of the gene with thin lines representing introns, thick lines representing coding exons [with the encoded Methionines (M) in green and other amino acids in blue], and intermediate thickness lines representing UTRs. For LRPPRC and PGK1, a zoomed out version is also shown in the dotted line box.

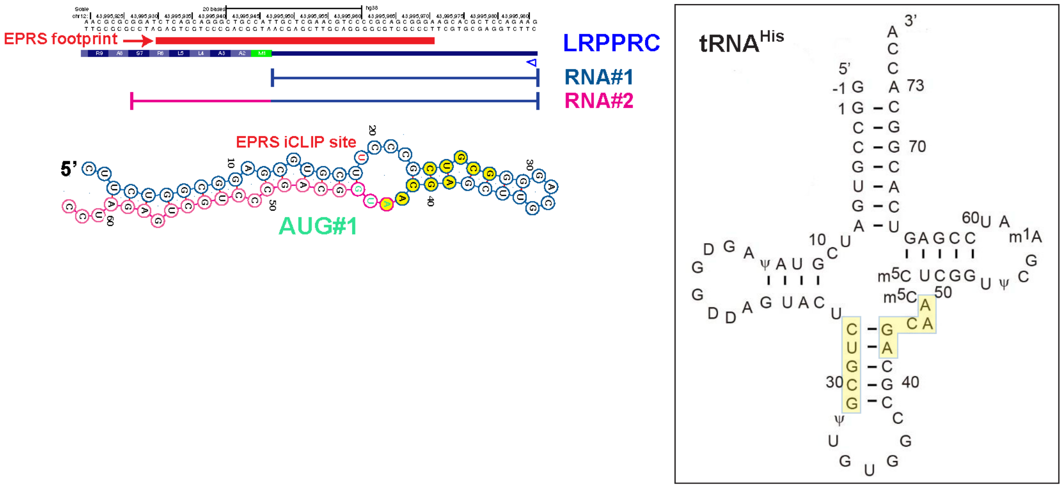

Appendix Fig S7. LRPPRC 5'UTR and tRNA^{His} share two identical 5-nt sequences.

Top Left, RNA#1 corresponds to the 5' UTR of LRPPRC (in teal color). RNA#2 corresponds to the 5' UTR of LRPPRC extended to the open reading frame (extension sequence shown in magenta).

Bottom Left: Predicted two-dimensional structure of RNA#2, with the sequence in common with RNA #1 shown in teal; the sequence unique to RNA#2 shown in magenta; EPRS-crosslinked site shown with red text; the start codon shown with green text; and the two 5-nt sequences identical to tRNA^{His} highlighted in yellow.

Right: Predicted two-dimensional structure of human tRNA^{His} with the two 5-nt sequences identical to LRPPRC 5'UTR highlighted in yellow.

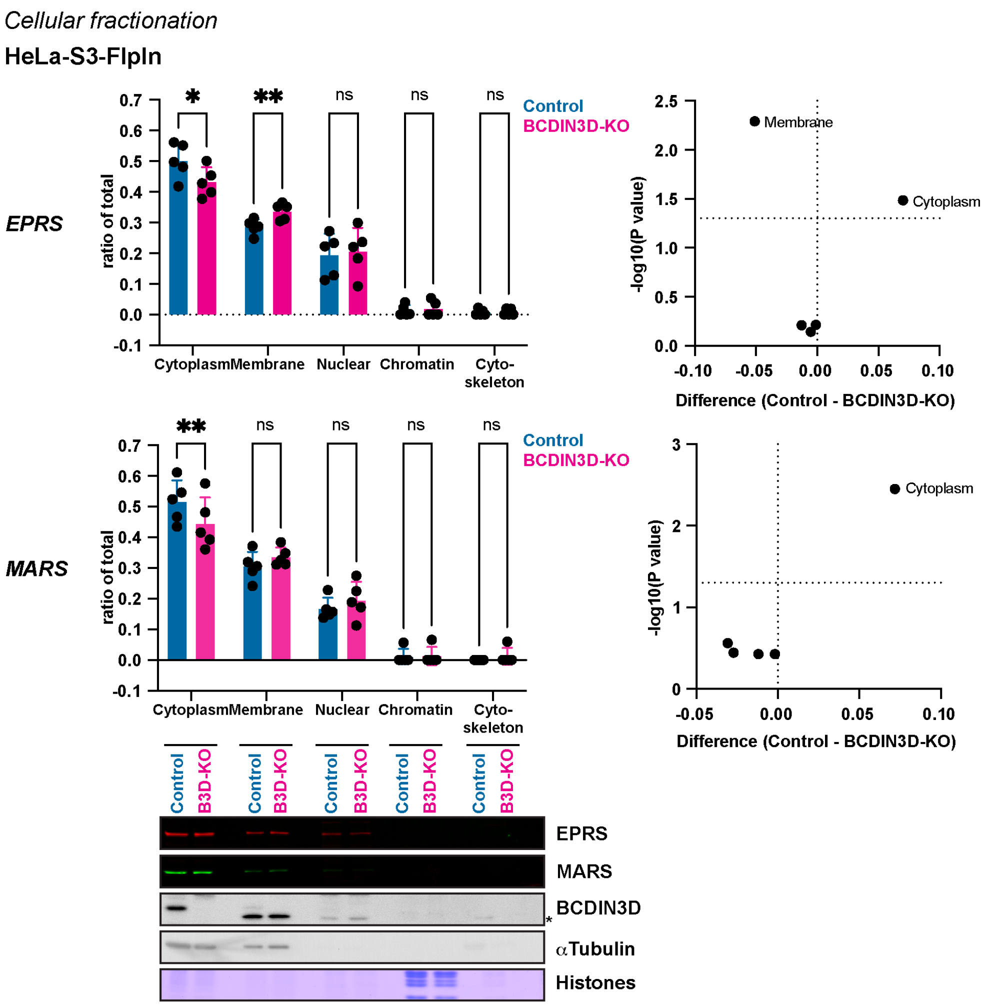

Appendix Fig S8. BCDIN3D knock out increases fraction of membrane-bound EPRS but not MARS.

Bottom: Representative quantitative LI-COR western blot of cellular fractions of HeLa-S3-FlpIn control and BCDIN3D-KO cells with antibodies against EPRS (red) and MARS (green). Shown are also western blots showing the fractionation of BCDIN3D and α-Tubulin, and Coomassie staining showing histones. Asterisk indicates a non-specific band detected by the BCDIN3D antibody.

Top: quantification as ratio of total of EPRS and MARS quantitative LI-COR western blots. Shown are mean±SD (n=5 biological replicates) and results of multiple paired t-tests without correction (see P value distribution on the volcano plots on the right), * p value < 0.05, ** p value < 0.01.

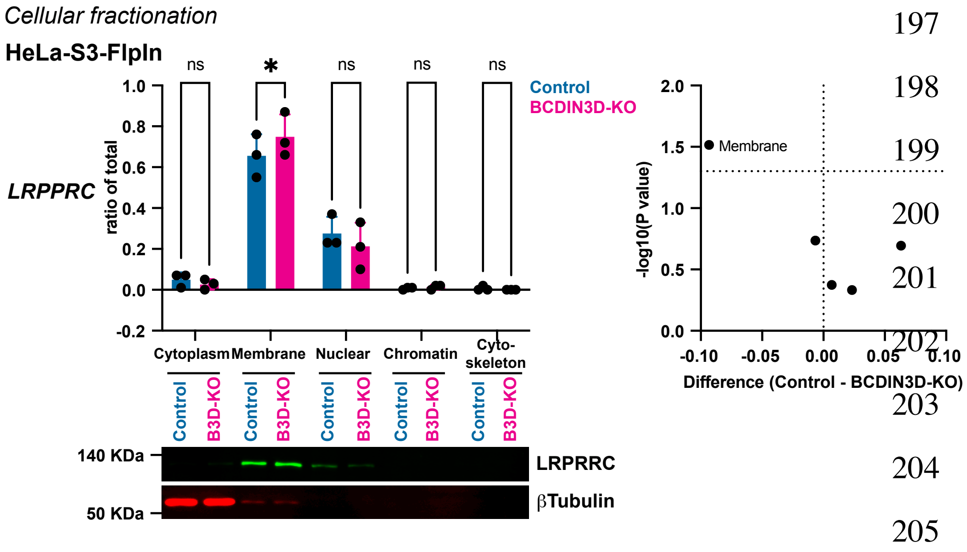

Appendix Fig S9. BCDIN3D knock out increases proportion of membrane-bound LRPPRC.

Top: Quantification as ratio of total of LRPPRC quantitative LI-COR western blots. Shown are mean±SD (n=3 biological replicates) and results of multiple paired t-tests without correction (see P value distribution on the volcano plot on the right), * p value < 0.05.

Bottom: Representative quantitative LI-COR western blots of cellular fractions of HeLa-S3-FlpIn control and BCDIN3D-KO cells with antibodies against LRPPRC (green) and β-Tubulin (red). For other controls, see Appendix Fig S7.

Supplemental References

Ceci M, Gaviraghi C, Gorrini C, Sala LA, Offenhauser N, Marchisio PC, Biffo S (2003) Release of eIF6 (p27BBP) from the 60S subunit allows 80S ribosome assembly. *Nature* 426: 579-584.
