# Supplementary figures and images for: ChemRAP uncovers specific mRNA translation regulation via RNA 5′ phospho-methylation
Source: EMBO Rep. 2024 Jan 23;25(3):1570–88. doi: 10.1038/s44319-024-00059-z (PMC10933402; doi:10.1038/s44319-024-00059-z)

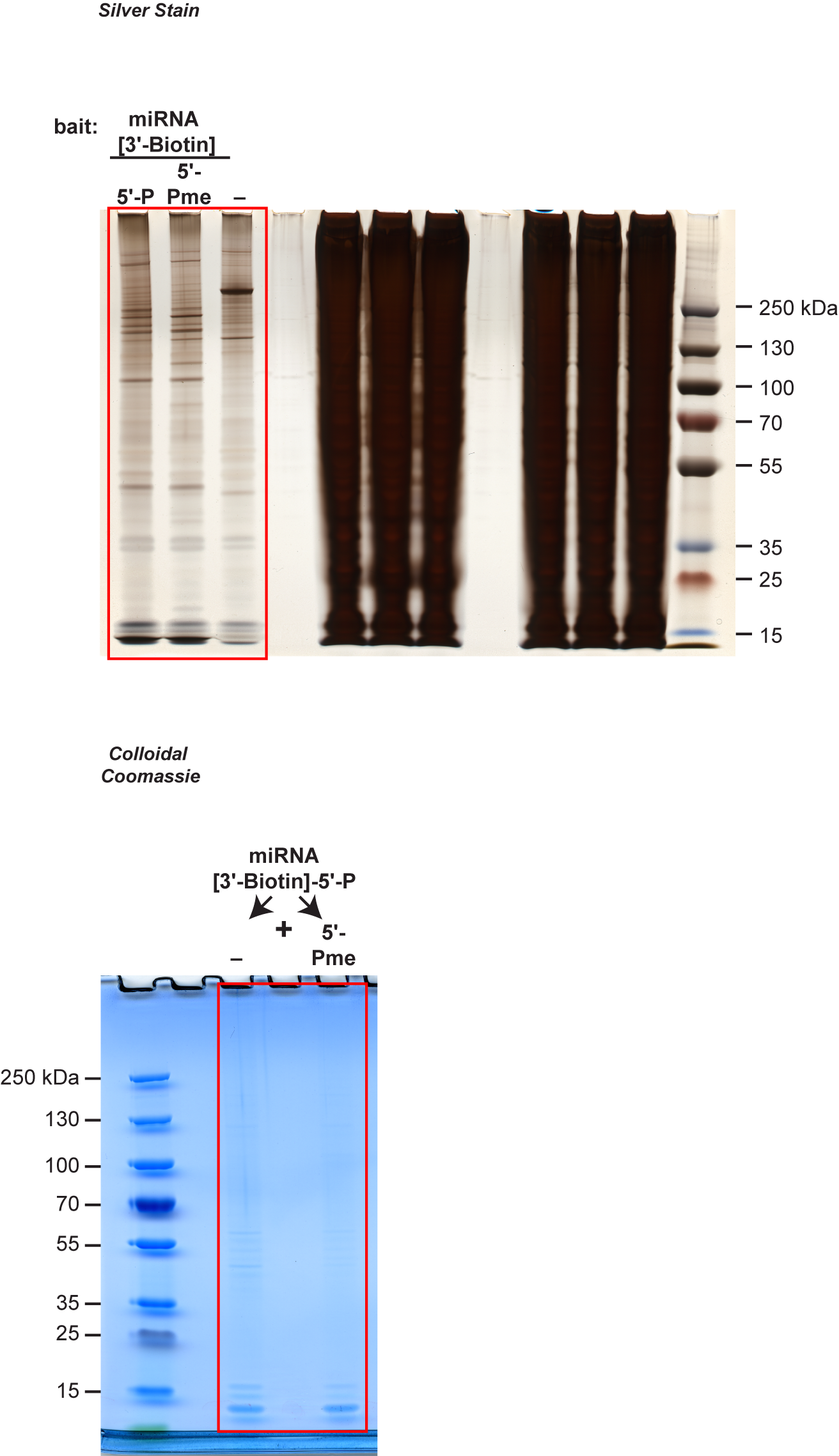

Supplement: Supplementary file 5 — Source Data Fig. 1 [file 44319_2024_59_MOESM5_ESM.zip › Figure 1/Figure 1B/Figure1B.tif]

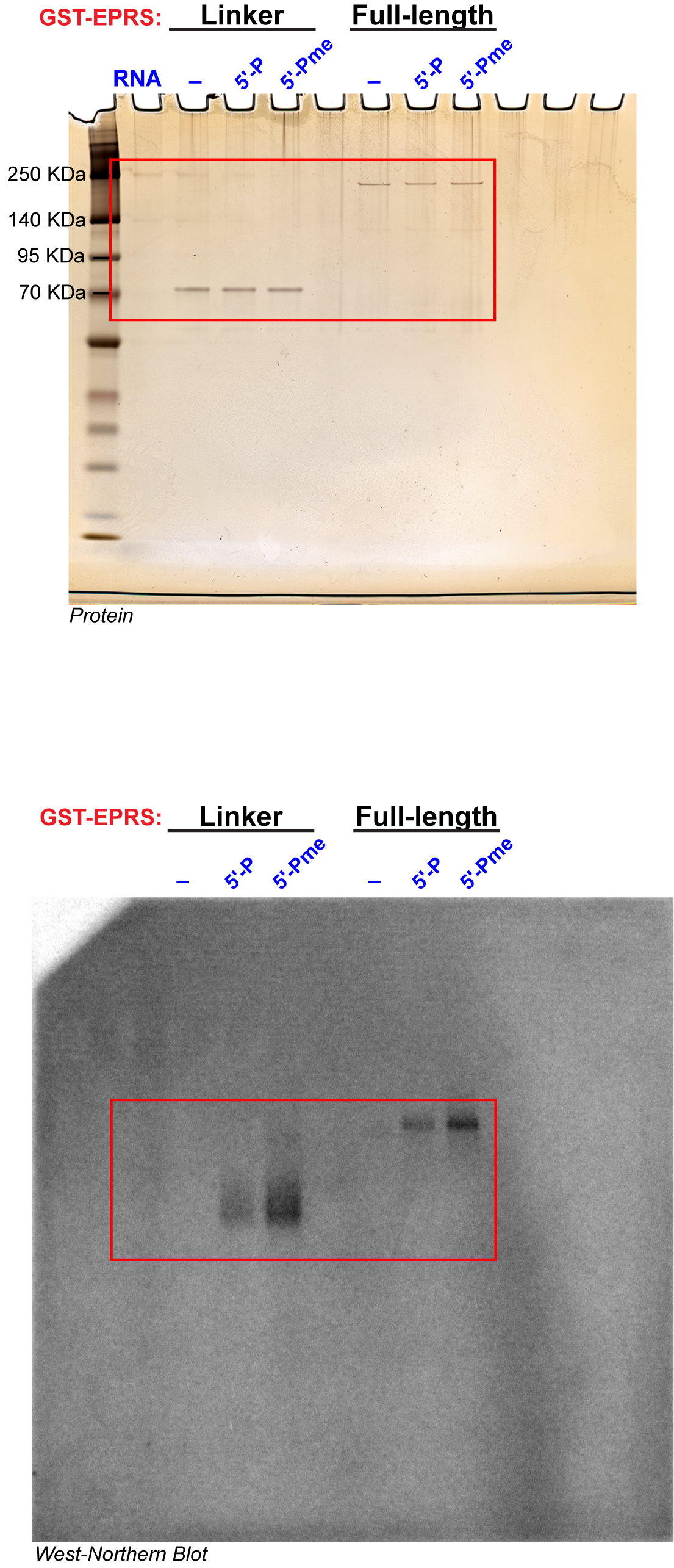

Supplement: Supplementary file 6 — Source Data Fig. 2 [file 44319_2024_59_MOESM6_ESM.zip › Figure 2/Figure 2F/Figure 2F.tif]

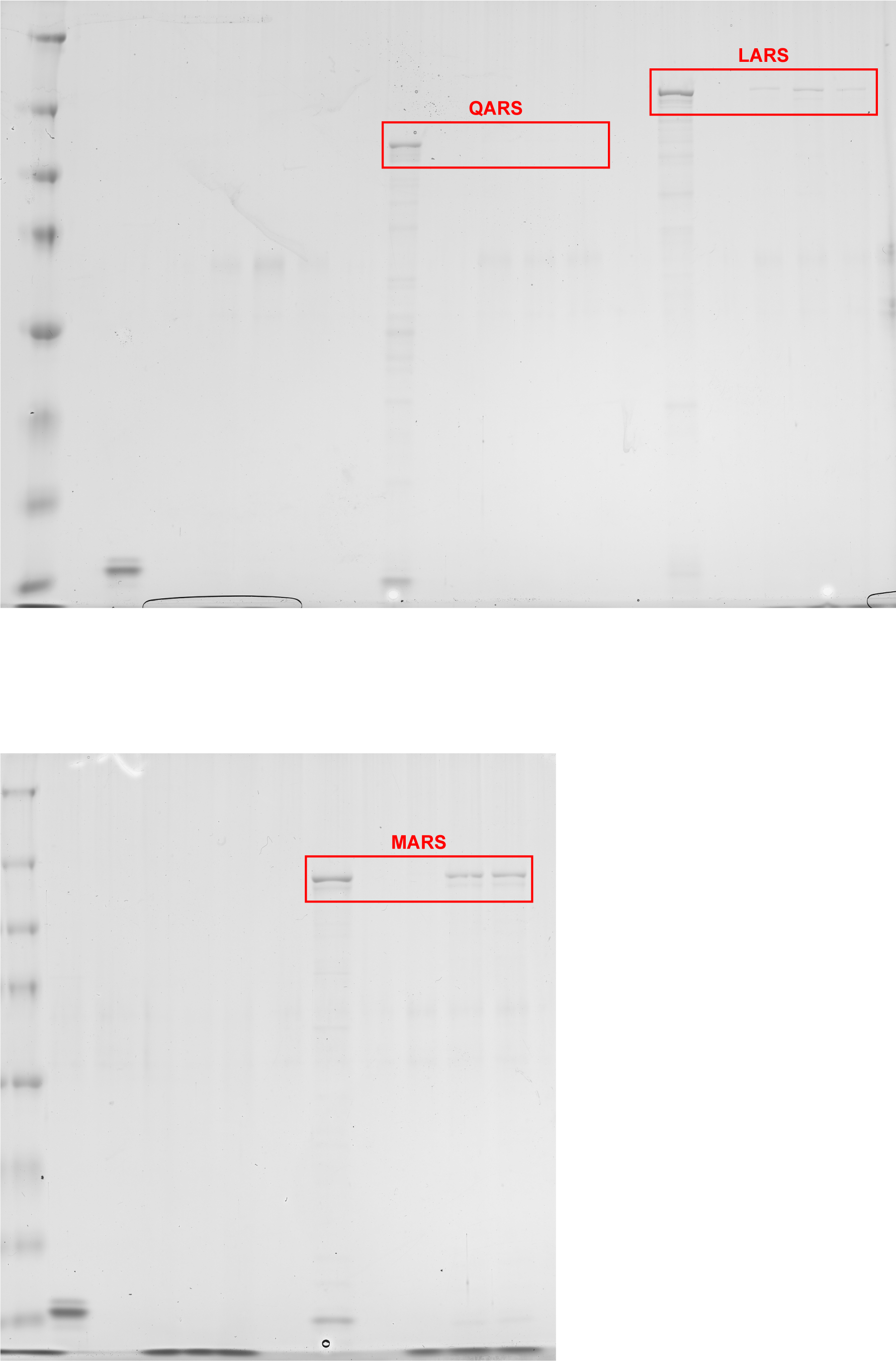

Supplement: Supplementary file 6 — Source Data Fig. 2 [file 44319_2024_59_MOESM6_ESM.zip › Figure 2/Figure 2C/Figure 2C_LARS-QARS-MARS.tif]

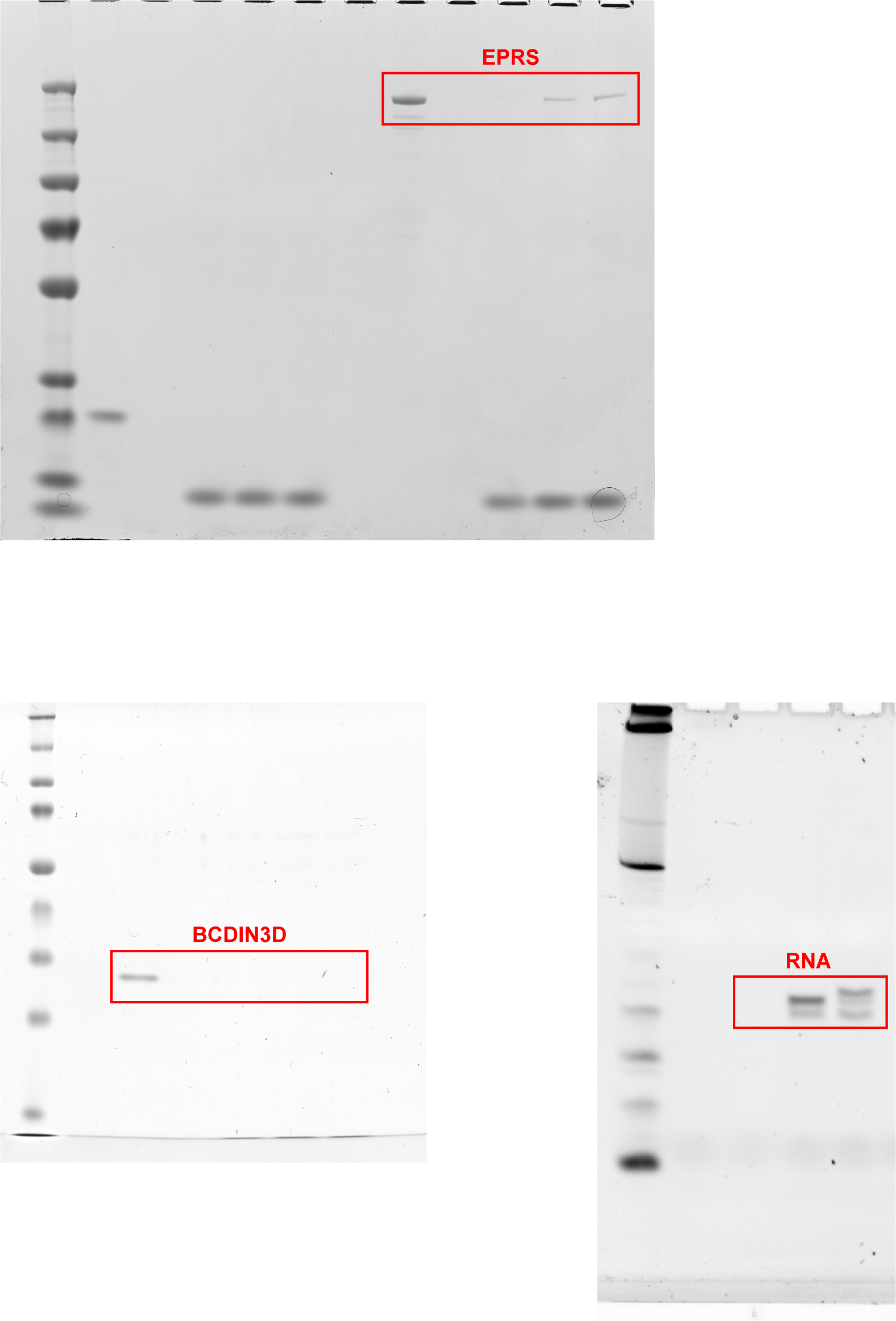

Supplement: Supplementary file 6 — Source Data Fig. 2 [file 44319_2024_59_MOESM6_ESM.zip › Figure 2/Figure 2C/Figure 2C_EPRS-BCDIN3D-miRNA.tif]

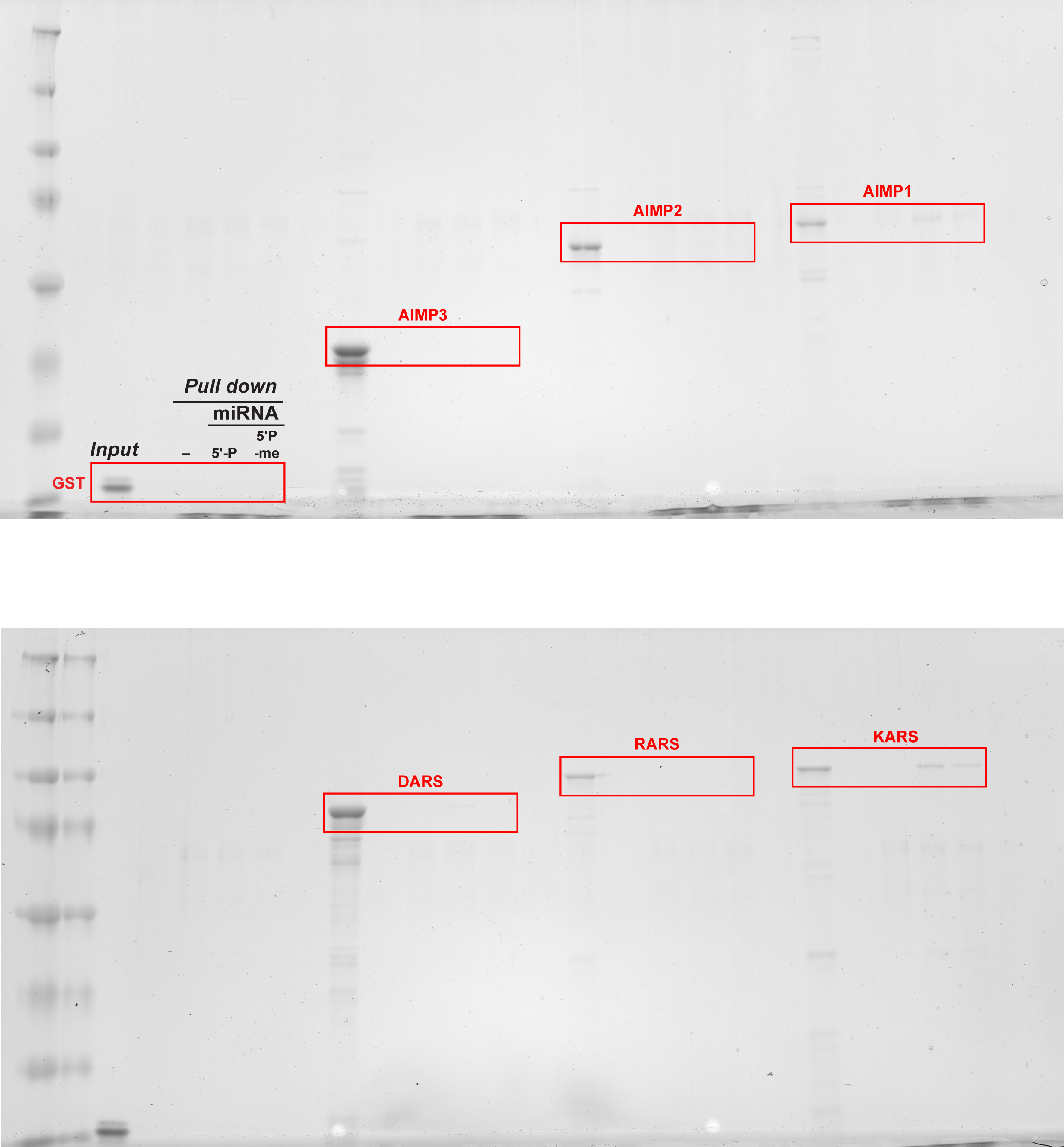

Supplement: Supplementary file 6 — Source Data Fig. 2 [file 44319_2024_59_MOESM6_ESM.zip › Figure 2/Figure 2C/Figure 2C_GST-AIMP1-AIMP2-AIMP3-DARS-RARS-KARS.tif]

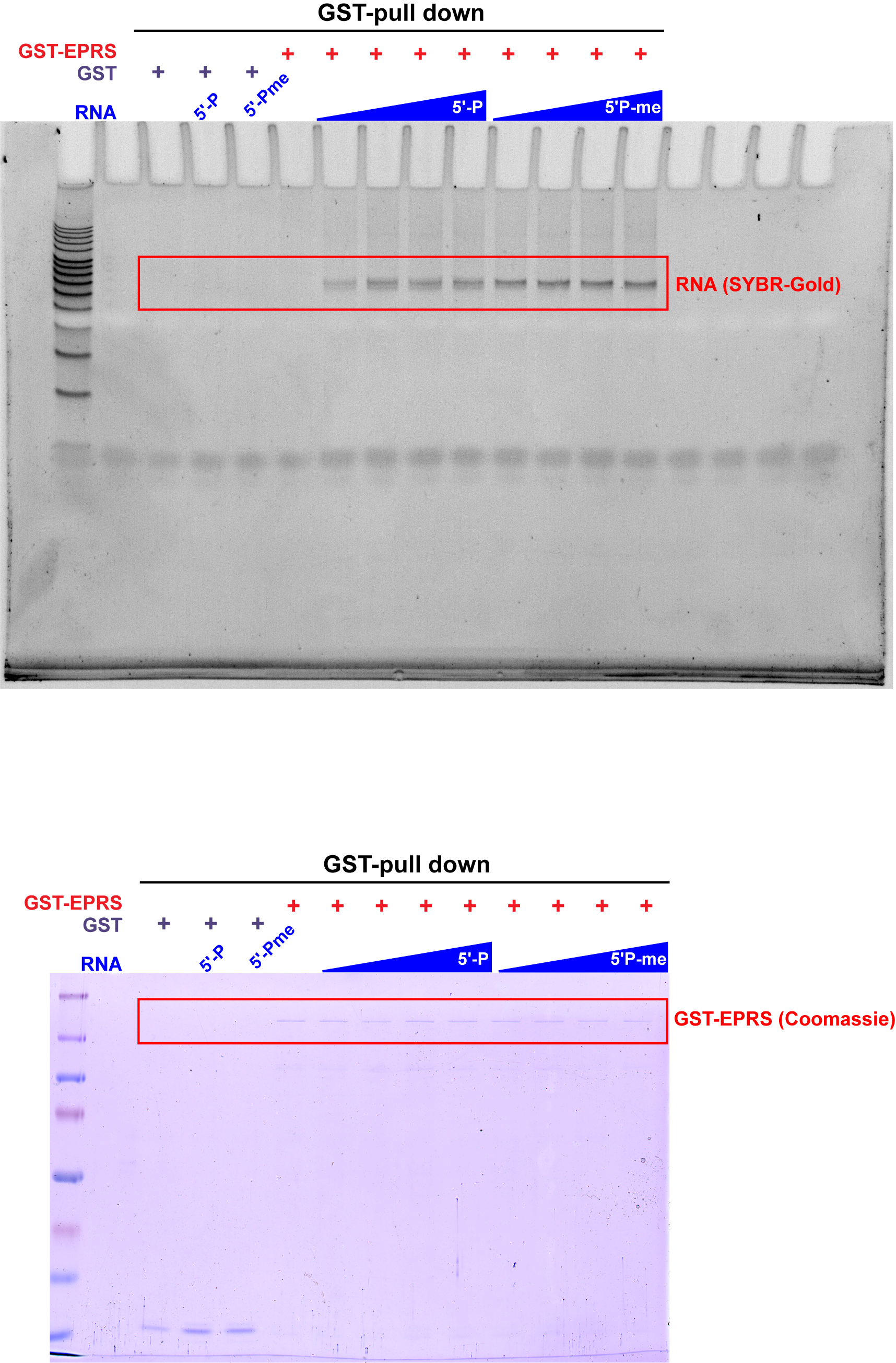

Supplement: Supplementary file 6 — Source Data Fig. 2 [file 44319_2024_59_MOESM6_ESM.zip › Figure 2/Figure 2D/Figure2D.tif]

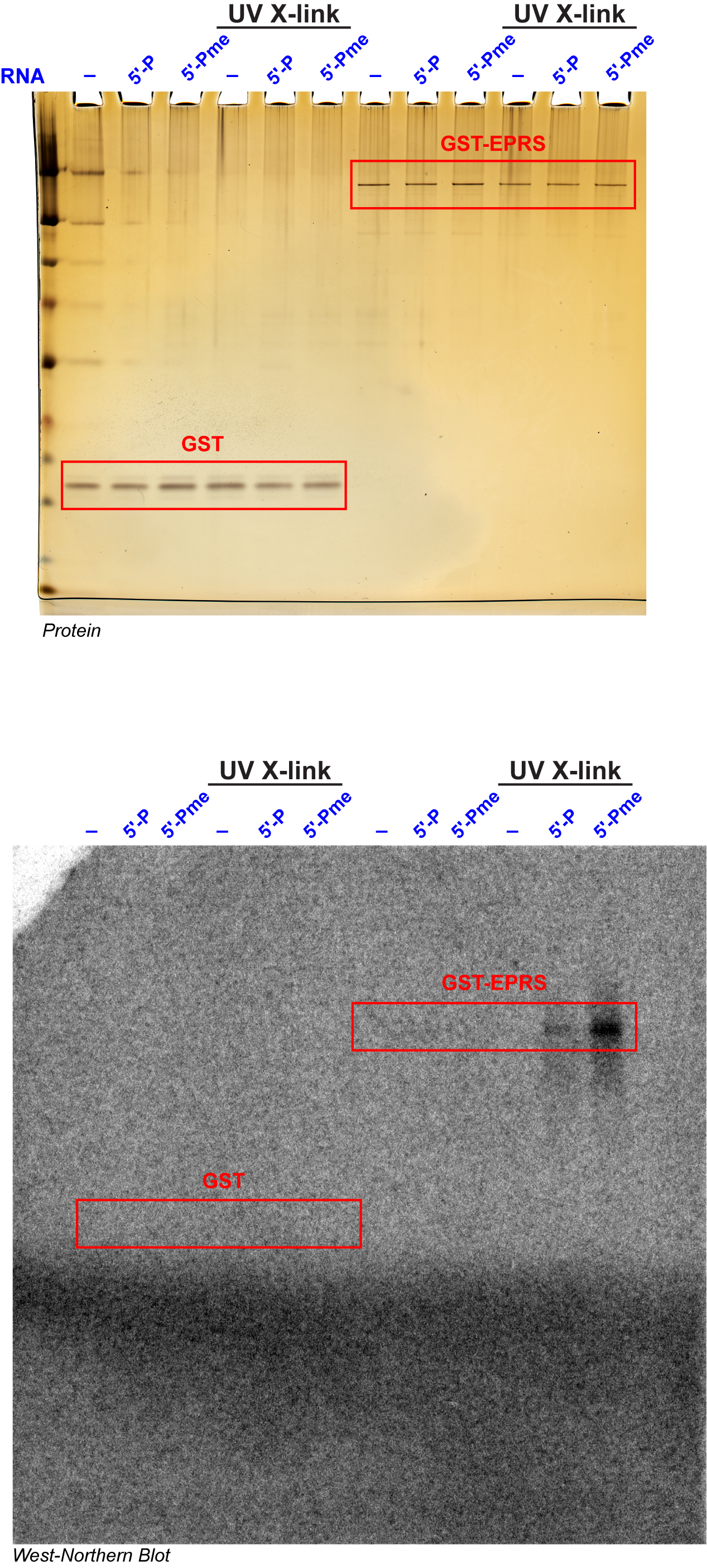

Supplement: Supplementary file 6 — Source Data Fig. 2 [file 44319_2024_59_MOESM6_ESM.zip › Figure 2/Figure 2E/Figure 2E.tif]

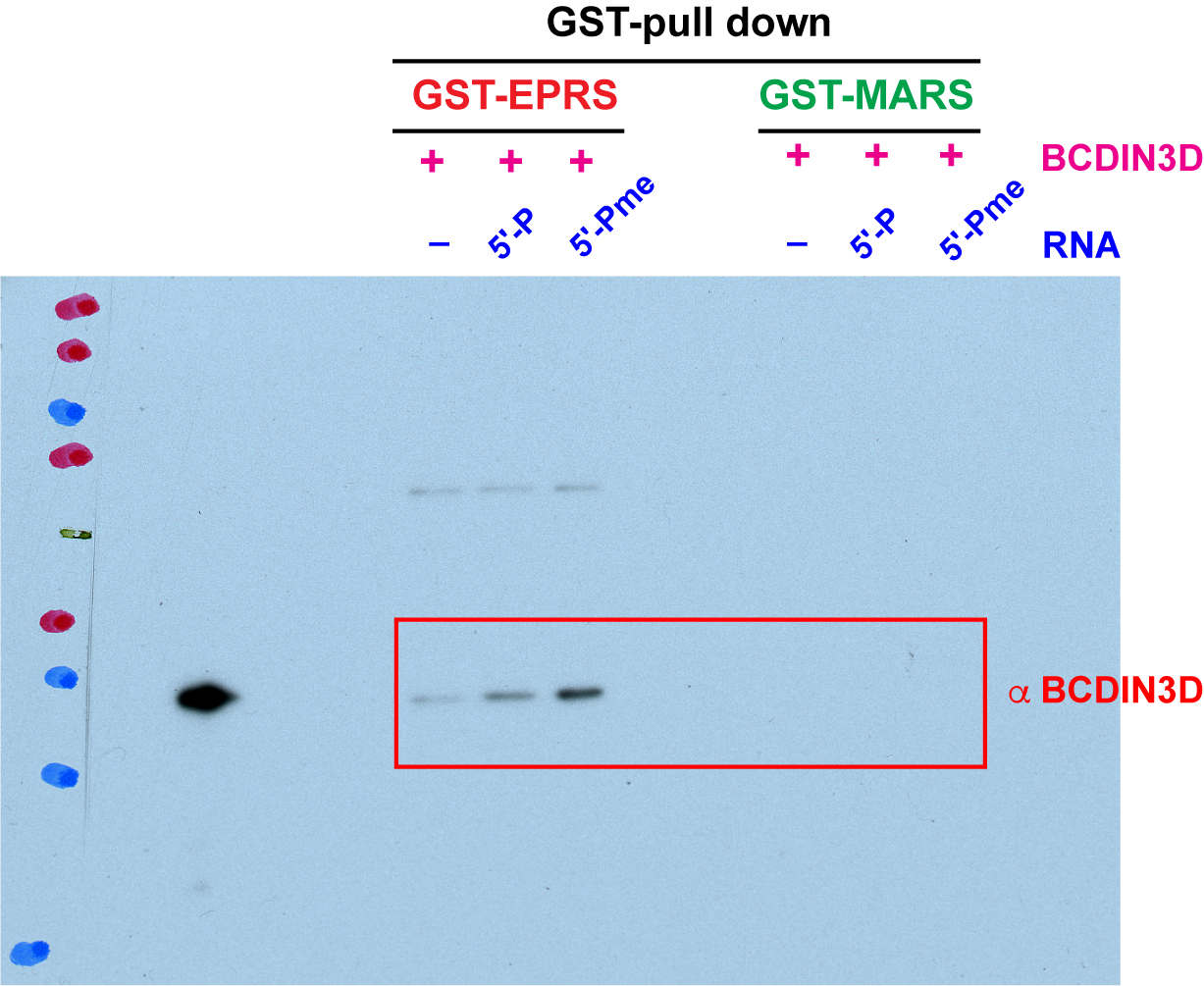

Supplement: Supplementary file 7 — Source Data Fig. 3 [file 44319_2024_59_MOESM7_ESM.zip › Figure 3/Figure 3B/Figure 3B.tif]

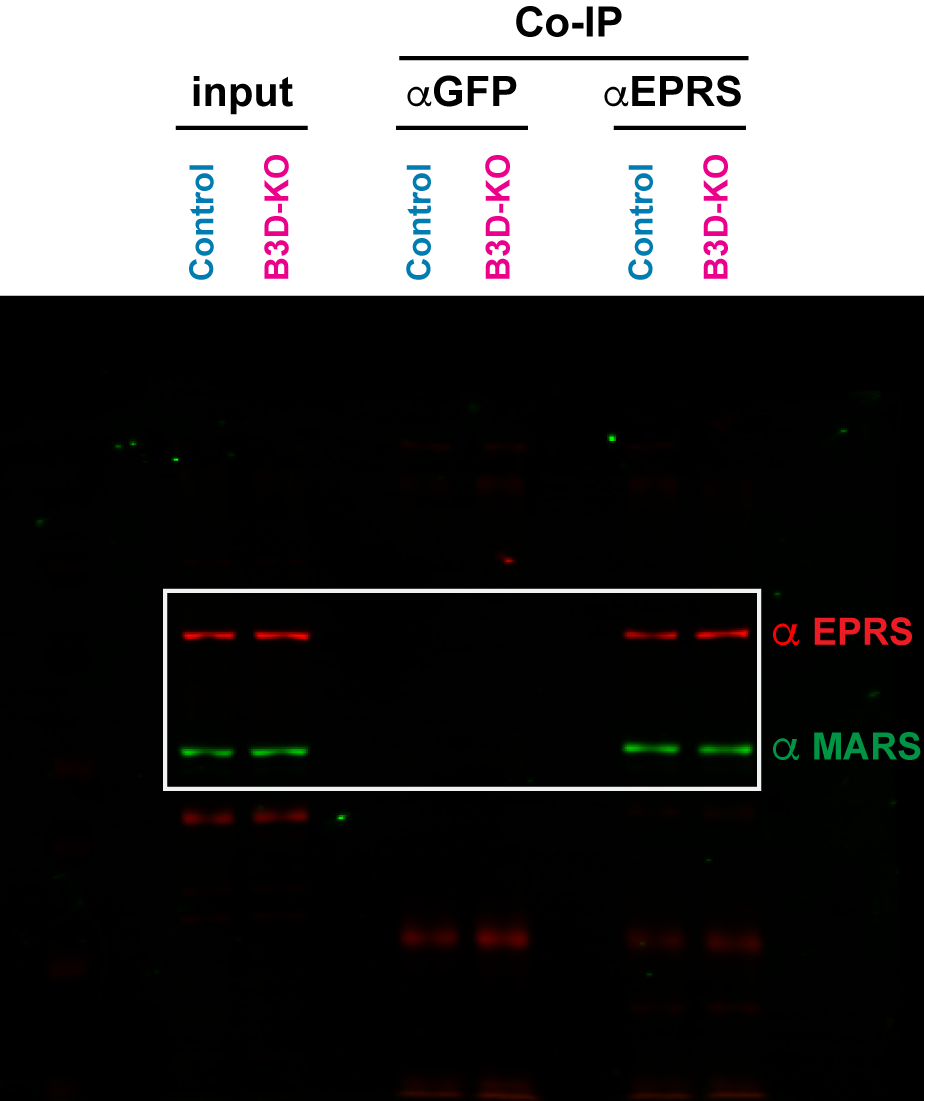

Supplement: Supplementary file 7 — Source Data Fig. 3 [file 44319_2024_59_MOESM7_ESM.zip › Figure 3/Figure 3D/Figure 3D.tif]

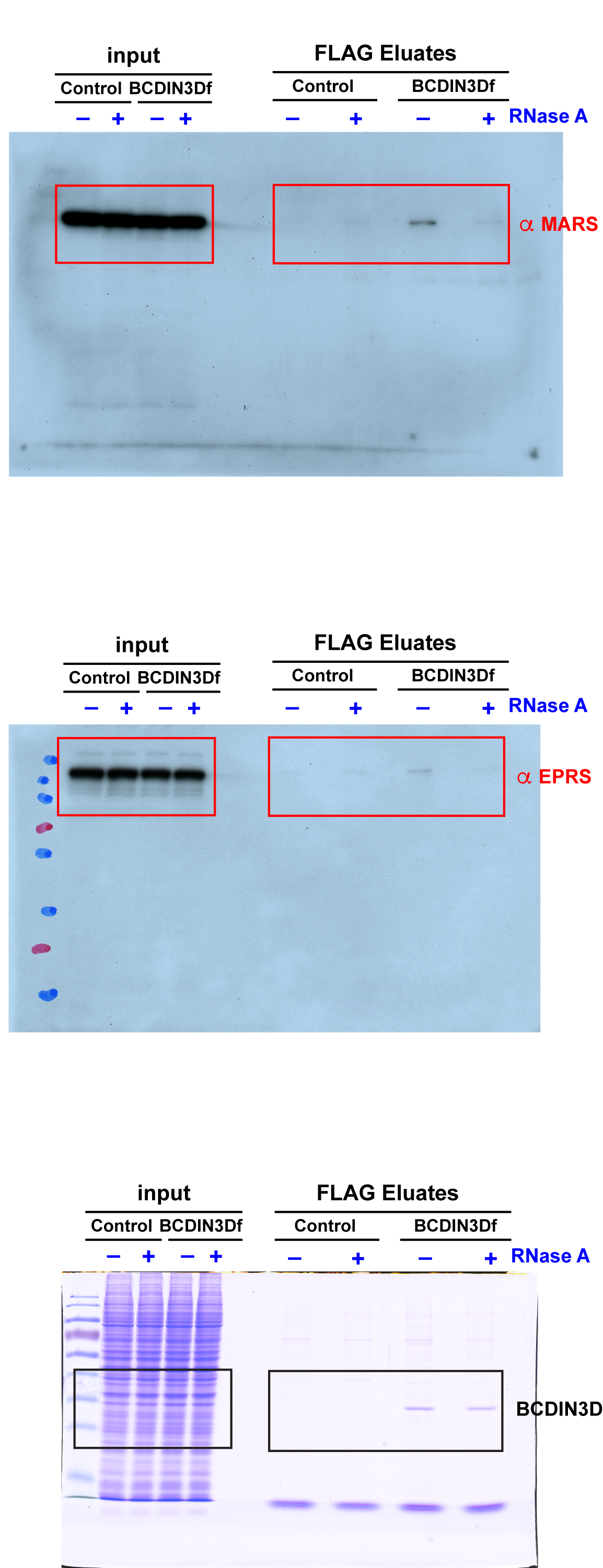

Supplement: Supplementary file 7 — Source Data Fig. 3 [file 44319_2024_59_MOESM7_ESM.zip › Figure 3/Figure 3A/Figure 3A.tif]

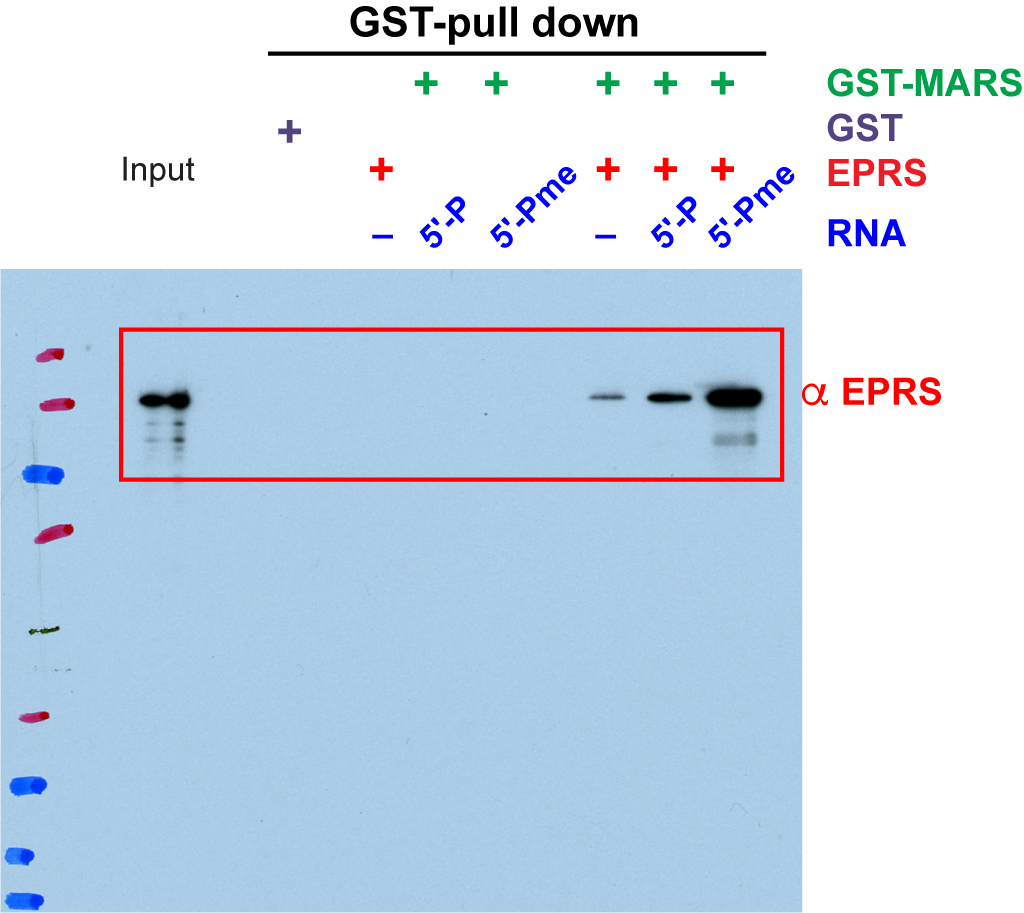

Supplement: Supplementary file 7 — Source Data Fig. 3 [file 44319_2024_59_MOESM7_ESM.zip › Figure 3/Figure 3F/Figure 3F.tif]

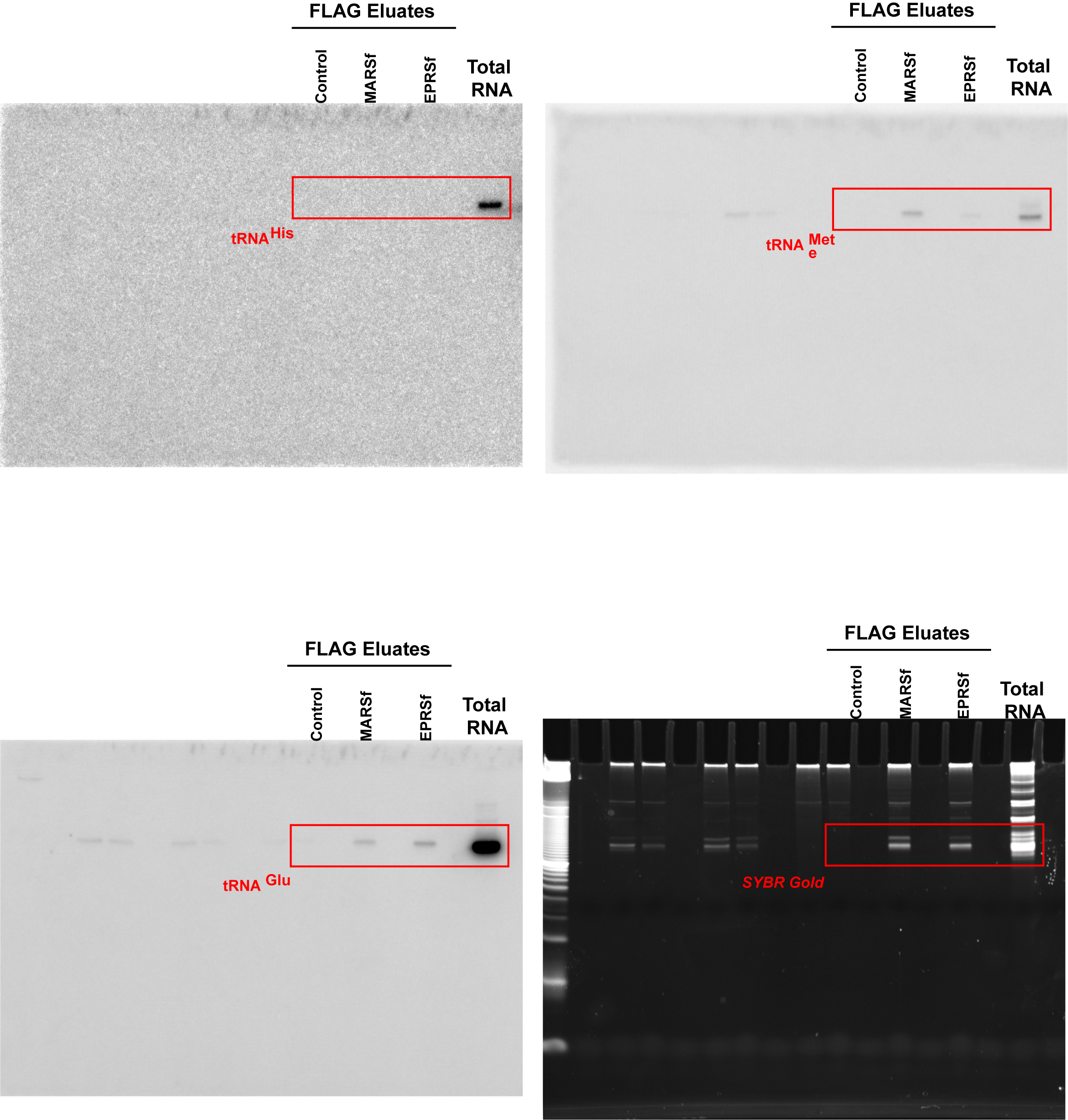

Supplement: Supplementary file 7 — Source Data Fig. 3 [file 44319_2024_59_MOESM7_ESM.zip › Figure 3/Figure 3G/Figure 3G.tif]

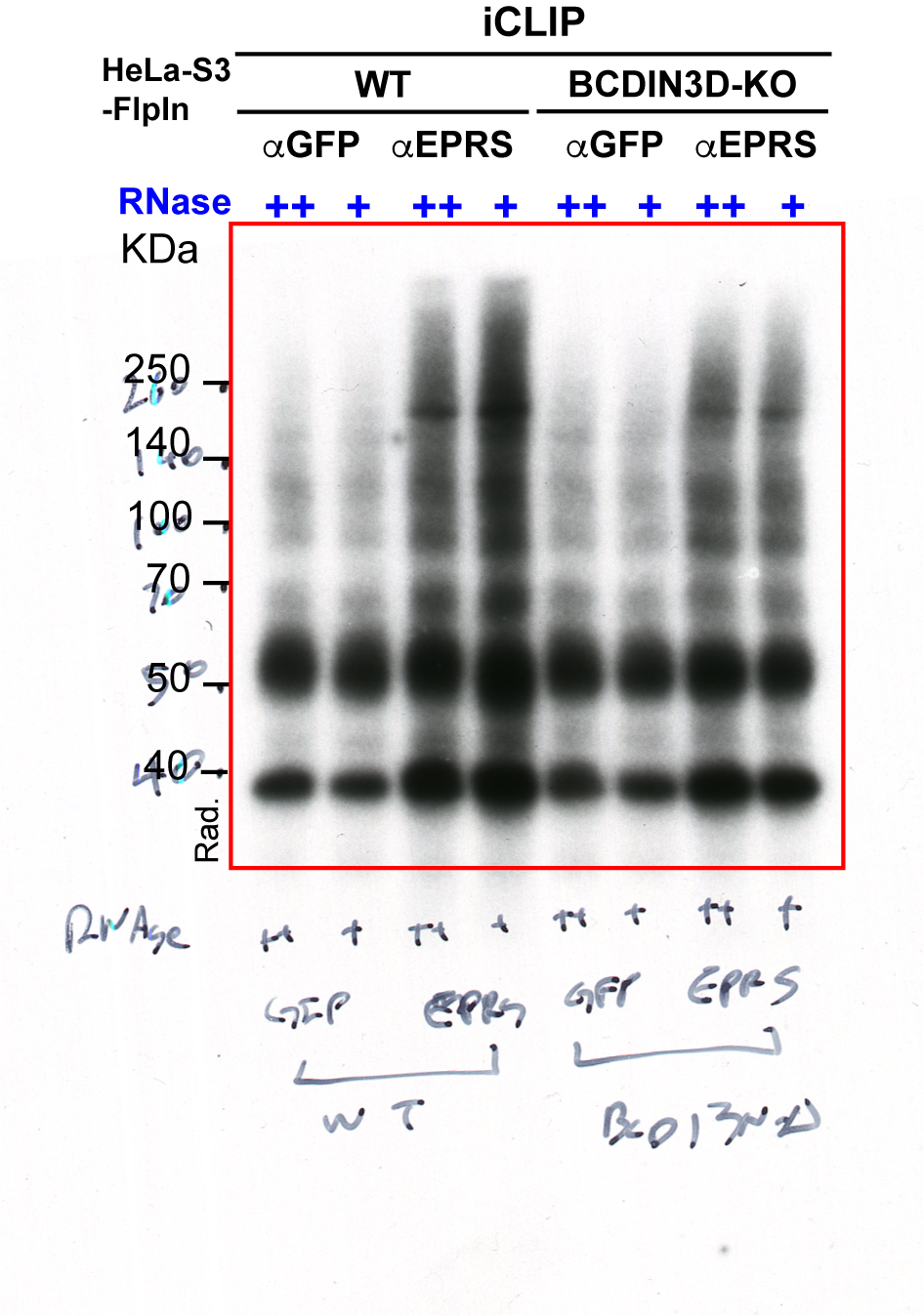

Supplement: Supplementary file 8 — Source Data Fig. 4 [file 44319_2024_59_MOESM8_ESM.zip › Figure 4/Figure 4A/Figure 4A.tif]

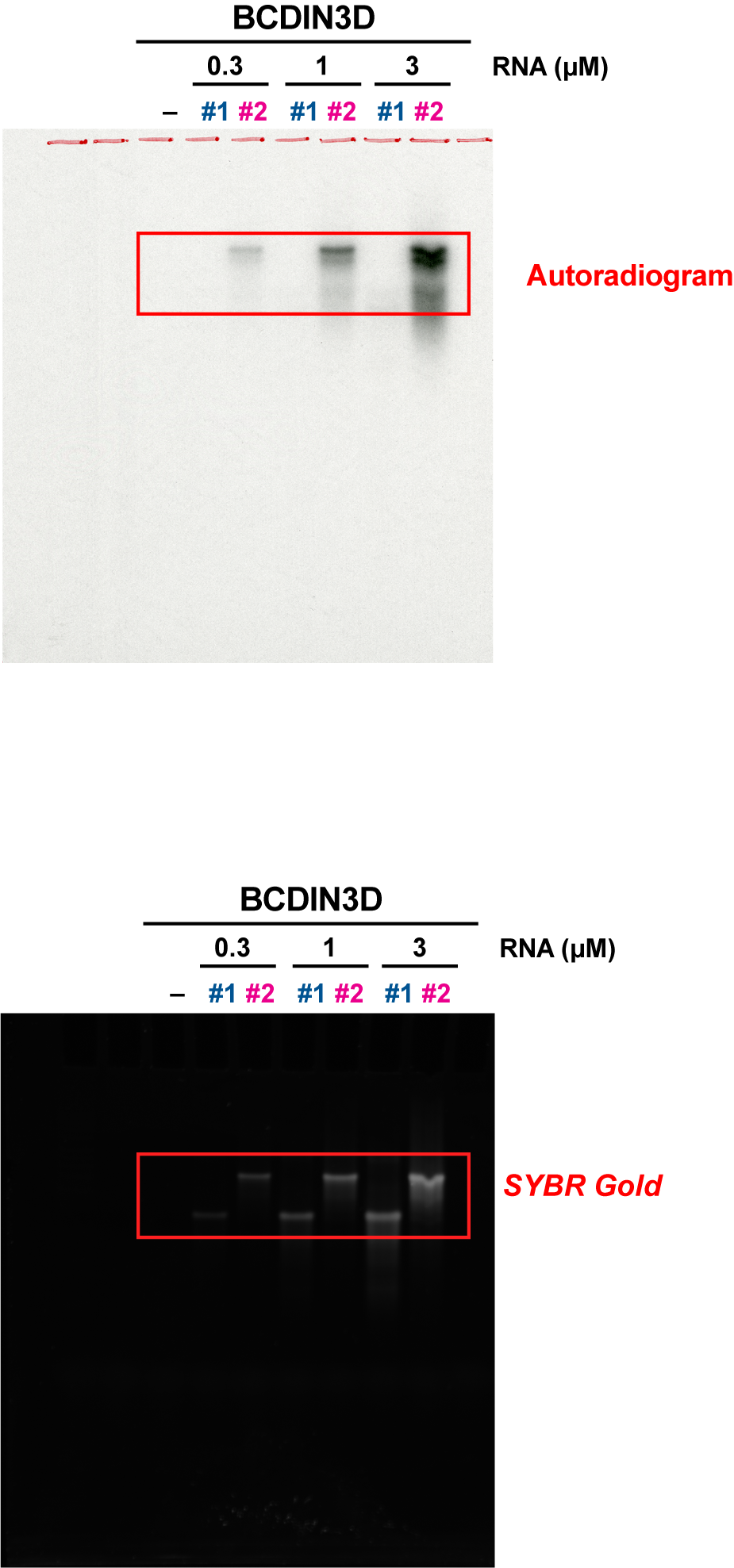

Supplement: Supplementary file 9 — Source Data Fig. 5 [file 44319_2024_59_MOESM9_ESM.zip › Figure 5/Figure 5D/Figure 5D.tif]

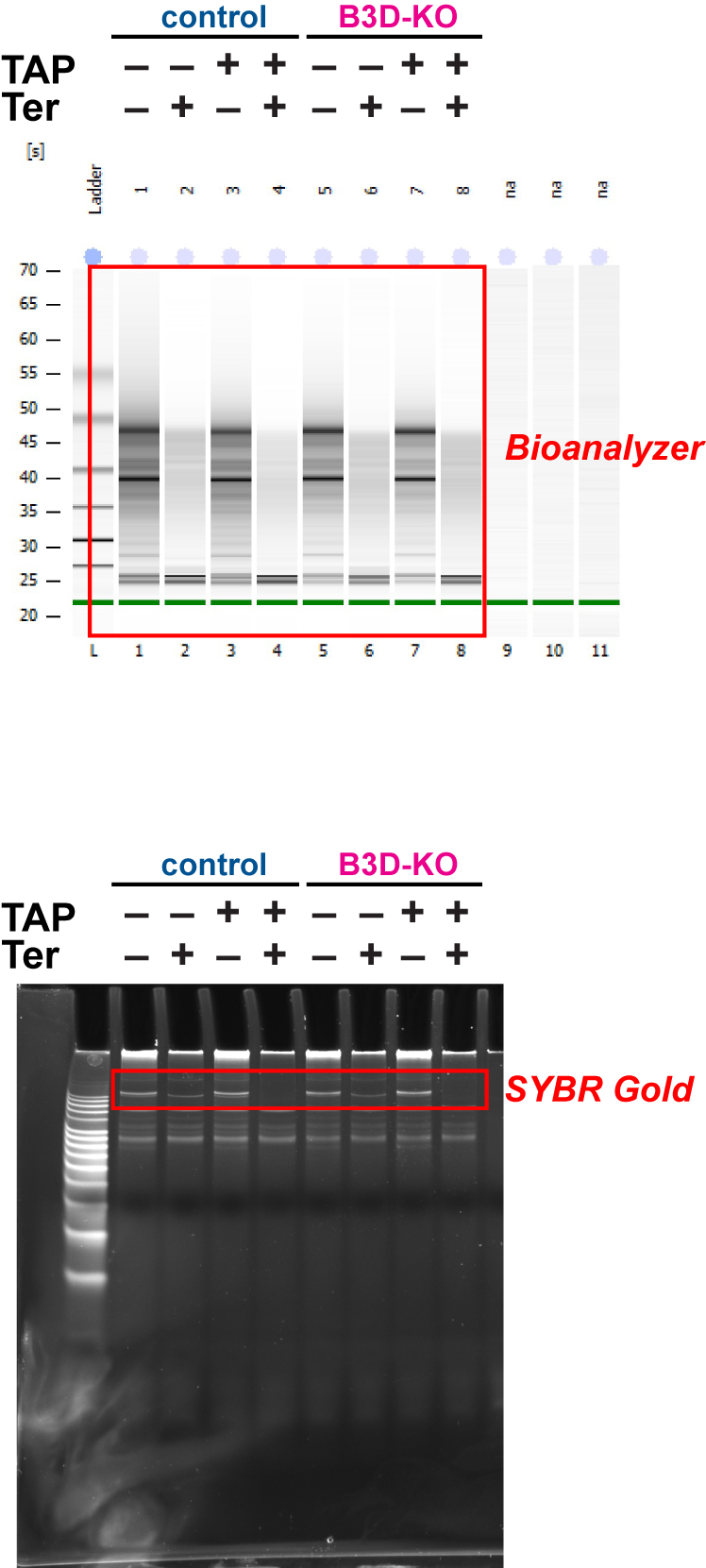

Supplement: Supplementary file 9 — Source Data Fig. 5 [file 44319_2024_59_MOESM9_ESM.zip › Figure 5/Figure 5B/Figure 5B.tif]

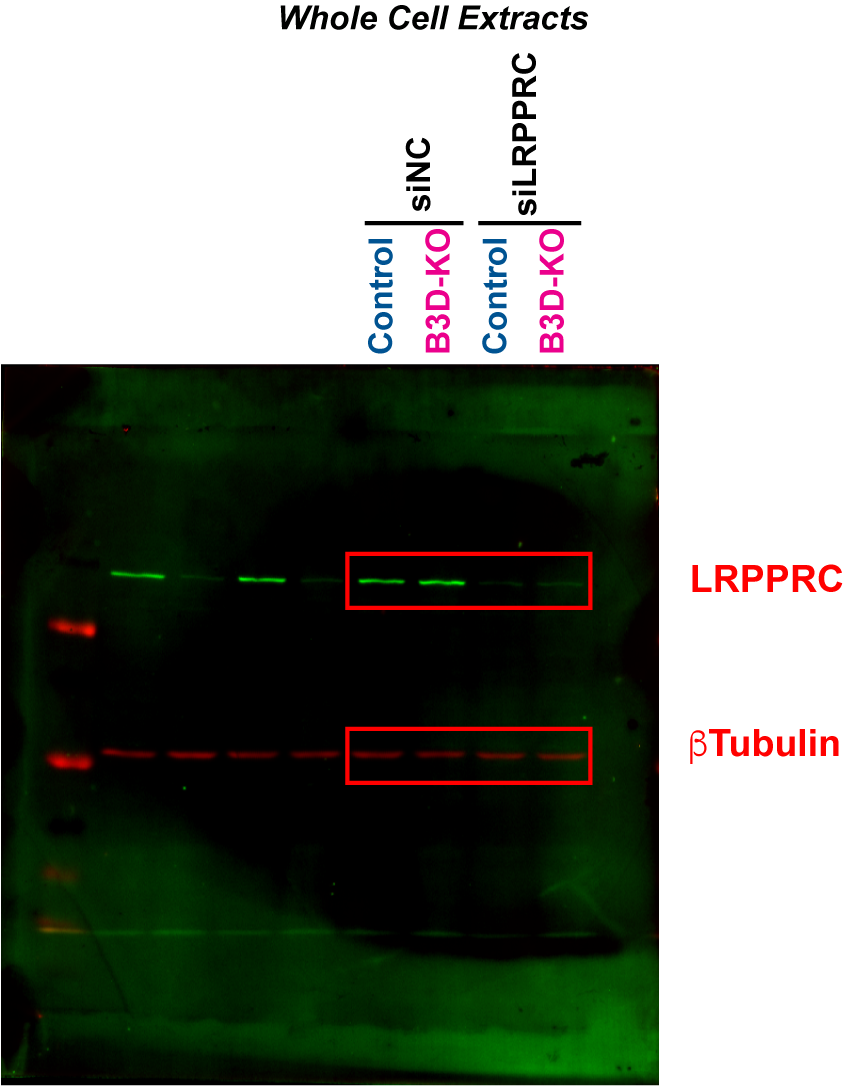

Supplement: Supplementary file 10 — Source Data Fig. 6 [file 44319_2024_59_MOESM10_ESM.zip › Figure 6/Figure 6A/Figure 6A.tif]

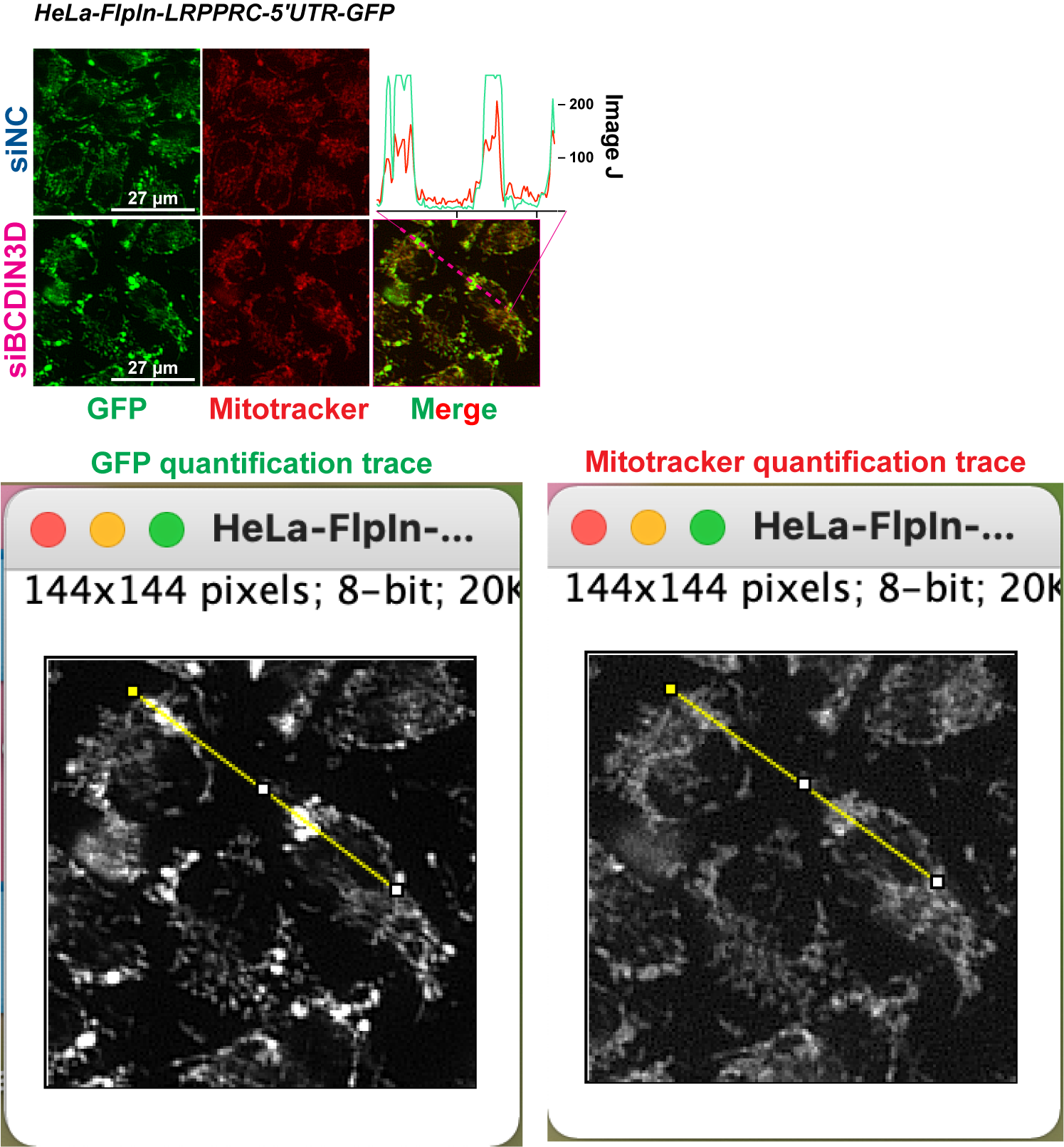

Supplement: Supplementary file 10 — Source Data Fig. 6 [file 44319_2024_59_MOESM10_ESM.zip › Figure 6/Figure 6I/Figure 6I.tif]

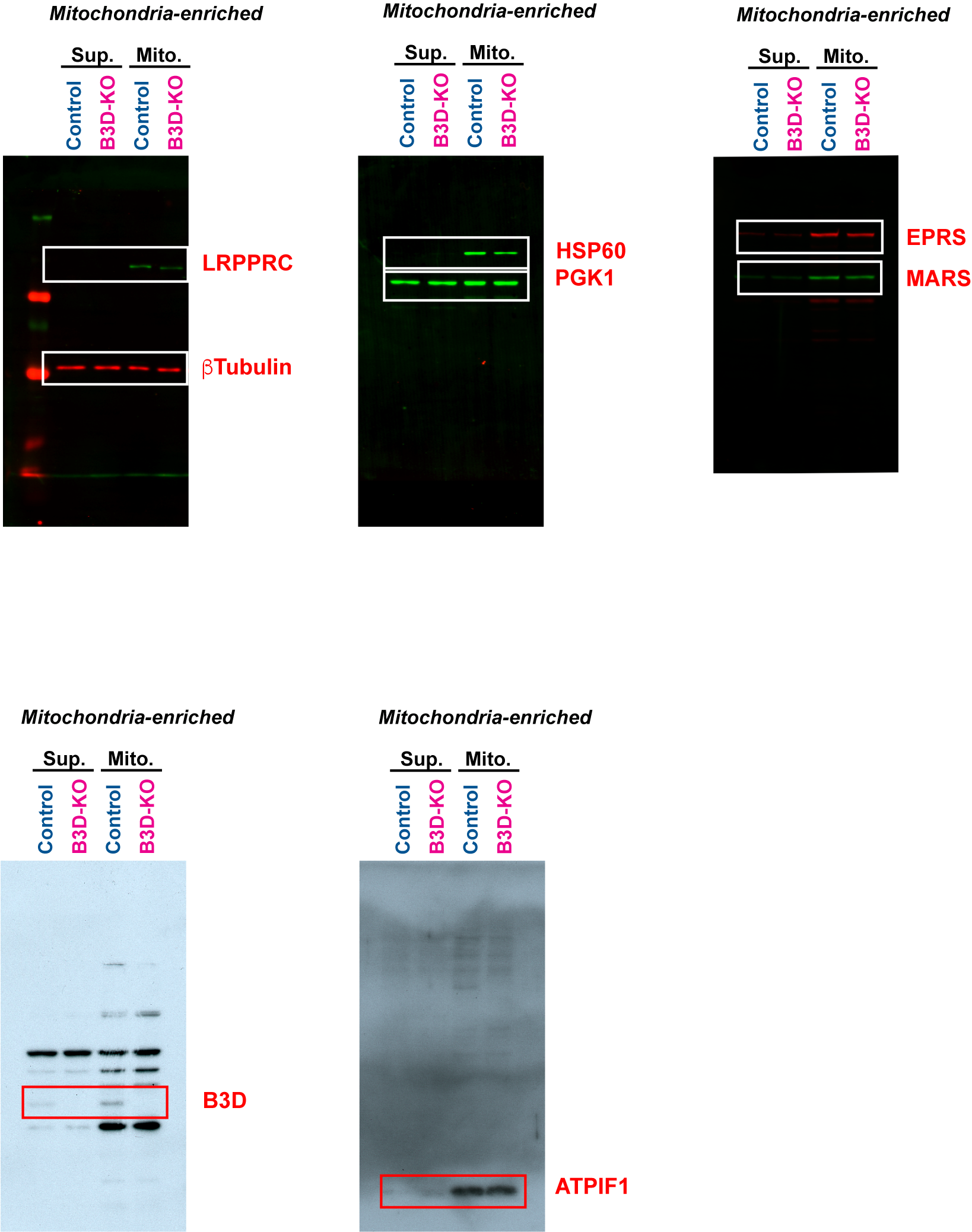

Supplement: Supplementary file 10 — Source Data Fig. 6 [file 44319_2024_59_MOESM10_ESM.zip › Figure 6/Figure 6G/Figure 6G.tif]

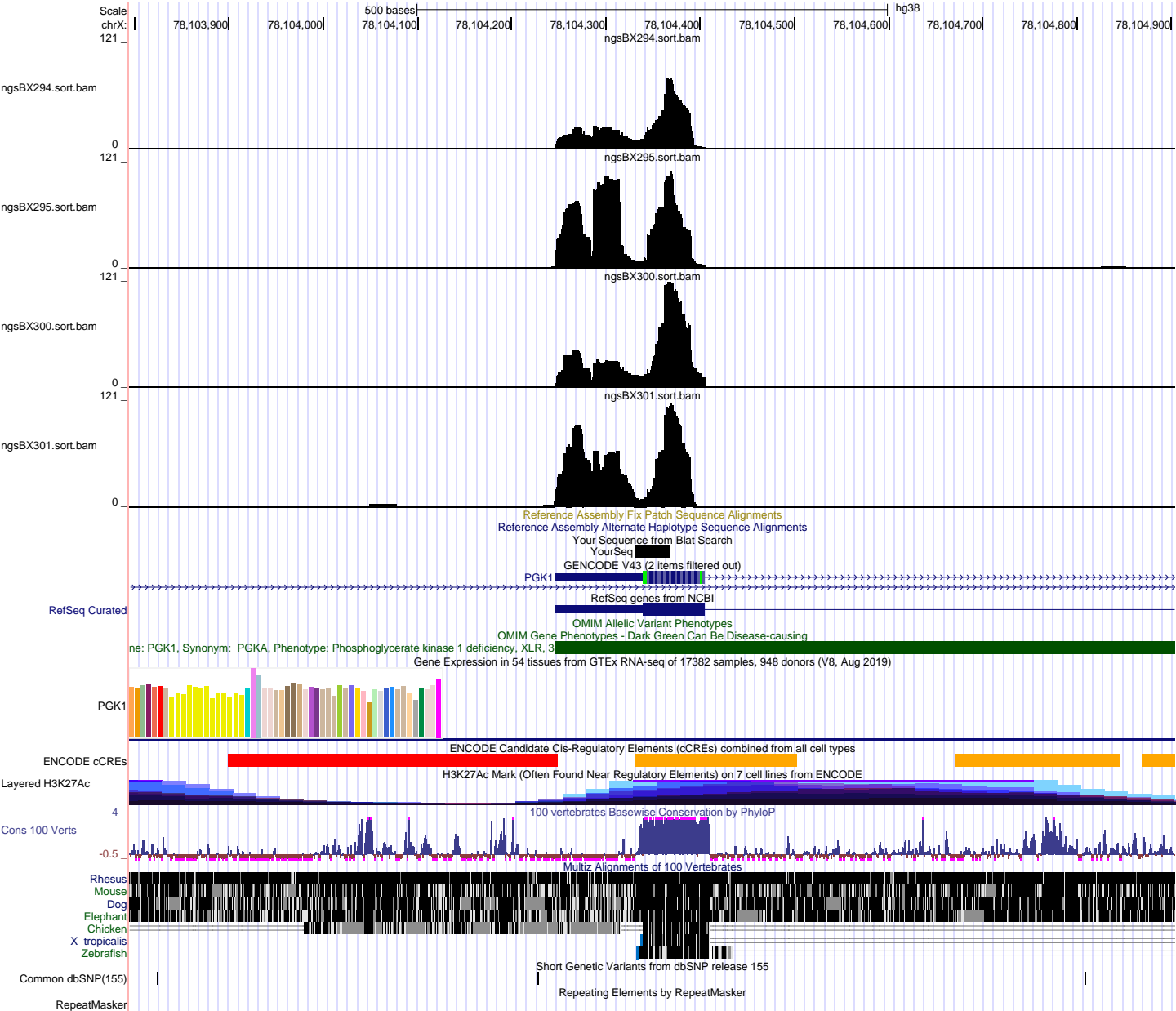

Supplement: Supplementary file 10 — Source Data Fig. 6 [file 44319_2024_59_MOESM10_ESM.zip › Figure 6/Figure 6D/Figure 6D_PGK1_hgt_genome_11451_d3e2e0.pdf]

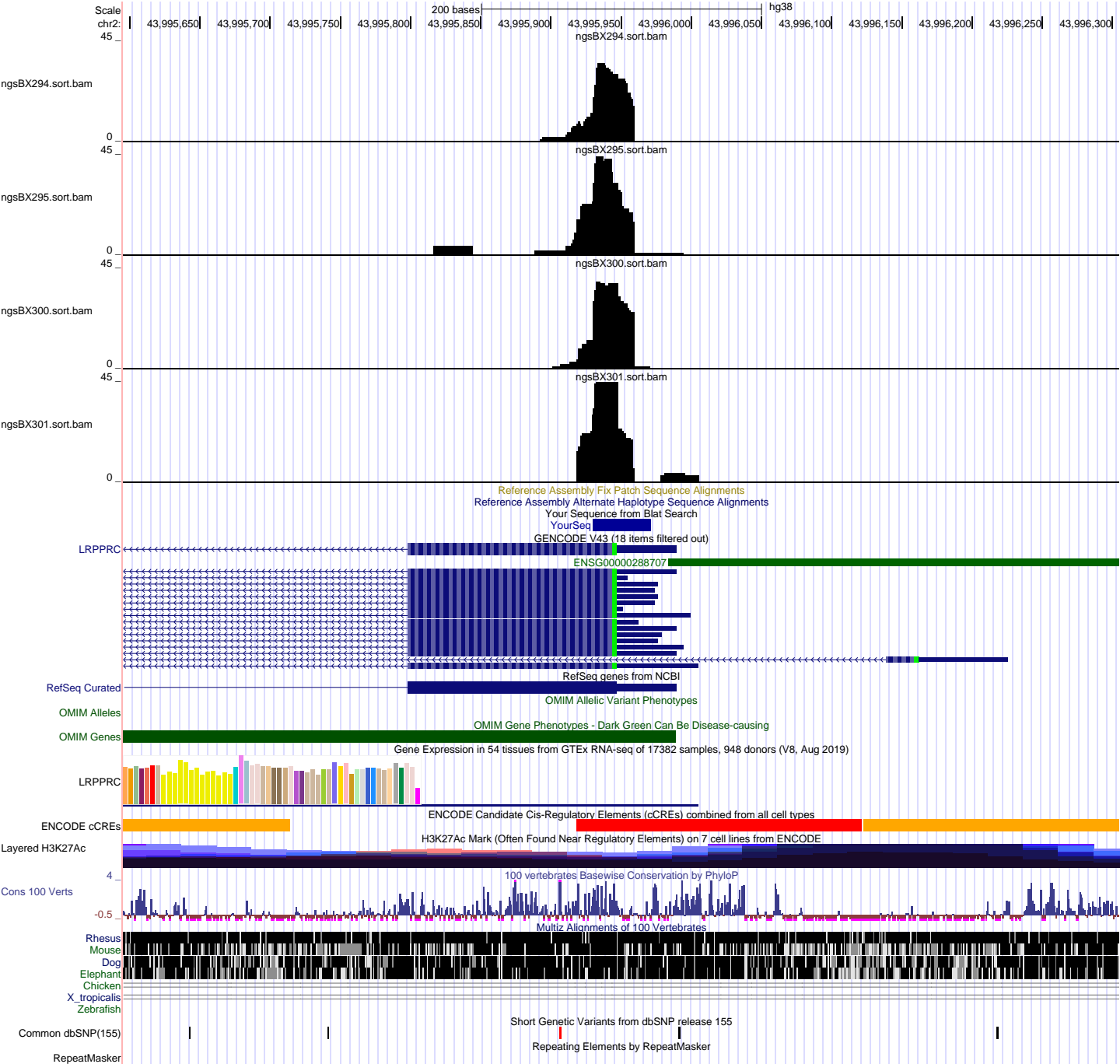

Supplement: Supplementary file 10 — Source Data Fig. 6 [file 44319_2024_59_MOESM10_ESM.zip › Figure 6/Figure 6D/Figure 6D_LRPPRC_hgt_genome_3090e_6be280.pdf]

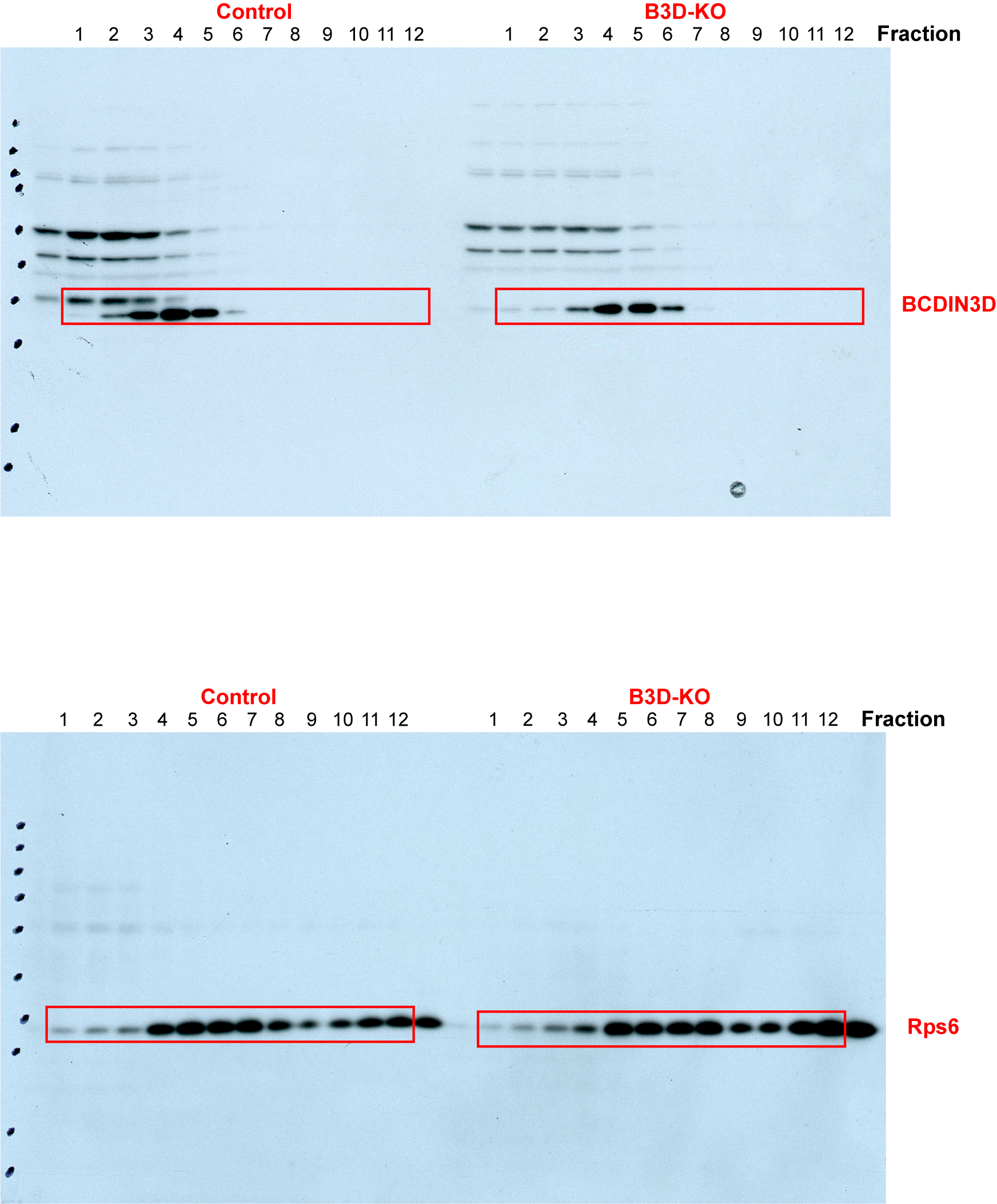

Supplement: Supplementary file 10 — Source Data Fig. 6 [file 44319_2024_59_MOESM10_ESM.zip › Figure 6/Figure 6C/Figure 6C_RPS6-BCDIN3D.tif]

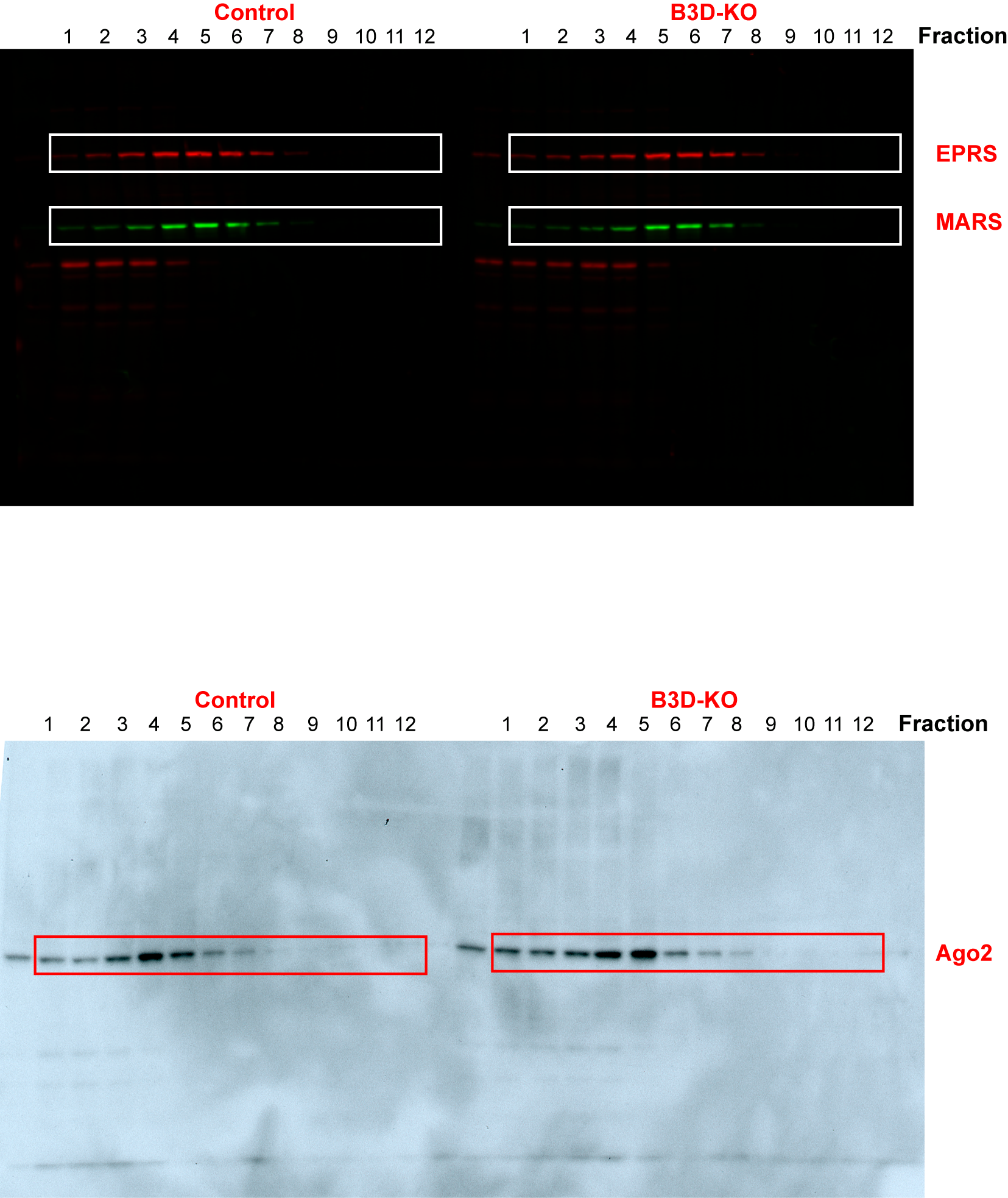

Supplement: Supplementary file 10 — Source Data Fig. 6 [file 44319_2024_59_MOESM10_ESM.zip › Figure 6/Figure 6C/Figure 6C_EPRS-MARS-Ago2.tif]
